# Abo1, a conserved bromodomain AAA‐ATPase, maintains global nucleosome occupancy and organisation

**DOI:** 10.15252/embr.201540476

**Published:** 2015-11-18

**Authors:** Csenge Gal, Heather E Murton, Lakxmi Subramanian, Alex J Whale, Karen M Moore, Konrad Paszkiewicz, Sandra Codlin, Jürg Bähler, Kevin M Creamer, Janet F Partridge, Robin C Allshire, Nicholas A Kent, Simon K Whitehall

**Affiliations:** ^1^ Institute for Cell & Molecular BiosciencesNewcastle UniversityNewcastleUK; ^2^ Wellcome Trust Centre for Cell Biology & Institute of Cell BiologyUniversity of EdinburghEdinburghUK; ^3^ Biosciences, College of Life & Environmental SciencesUniversity of ExeterExeterUK; ^4^ Department of Genetics, Evolution & Environment and UCL Cancer InstituteUniversity College LondonLondonUK; ^5^ Department of PathologySt. Jude Children's Research HospitalMemphisTNUSA; ^6^ Cardiff School of BiosciencesCardiff UniversityCardiffUK

**Keywords:** Abo1, bromodomain AAA‐ATPases, chromatin, nucleosome mapping, *Schizosaccharomyces pombe*, Chromatin, Epigenetics, Genomics & Functional Genomics

## Abstract

Maintenance of the correct level and organisation of nucleosomes is crucial for genome function. Here, we uncover a role for a conserved bromodomain AAA‐ATPase, Abo1, in the maintenance of nucleosome architecture in fission yeast. Cells lacking *abo1* ^+^ experience both a reduction and mis‐positioning of nucleosomes at transcribed sequences in addition to increased intragenic transcription, phenotypes that are hallmarks of defective chromatin re‐establishment behind RNA polymerase II. Abo1 is recruited to gene sequences and associates with histone H3 and the histone chaperone FACT. Furthermore, the distribution of Abo1 on chromatin is disturbed by impaired FACT function. The role of Abo1 extends to some promoters and also to silent heterochromatin. Abo1 is recruited to pericentromeric heterochromatin independently of the HP1 ortholog, Swi6, where it enforces proper nucleosome occupancy. Consequently, loss of Abo1 alleviates silencing and causes elevated chromosome mis‐segregation. We suggest that Abo1 provides a histone chaperone function that maintains nucleosome architecture genome‐wide.

## Introduction

Nuclear genomes are organised by assembly into chromatin. The fundamental repeating unit of chromatin is the nucleosome, which is composed of approximately 147 bp of DNA wrapped around an octamer of histone subunits. Nucleosomes represent an obstacle to DNA‐dependent processes such as transcription, and as a result, key steps in transcription involve regulated nucleosome removal and replacement 1. Transcription elongation by RNA polymerase II (RNAP II) may cause the displacement of H2A–H2B dimers from nucleosomes or even the eviction of entire histone octamers 1, 2, 3, 4. Therefore, RNAP II‐coupled nucleosome assembly is required to maintain the chromatin structure over gene bodies. A variety of factors have been shown to contribute to this process but prominent amongst them is FACT, an ATP‐independent histone chaperone complex that both facilitates nucleosome disassembly ahead of RNAP II and promotes reassembly behind it 5. Defects in transcription‐coupled chromatin assembly pathways result in a global decrease in nucleosome occupancy which leads to increased spurious intragenic transcription initiation 1.

Human ATAD2 (or ANCCA) is an evolutionarily conserved bromodomain containing ATPase that functions as a transcriptional co‐regulator via the control of chromatin 6, 7, 8, 9, 10. ATAD2 is predominantly expressed in the male germline but is systematically over‐expressed in tumours and is significantly associated with lung and breast cancers with poor prognoses 8, 11, 12, 13. A paralogous protein, ATAD2B, has been shown to be expressed during neuronal differentiation 14, but its molecular functions have not been determined. ATAD2 and ATAD2B belong to the AAA (ATPases associated with diverse cellular activities) ATPase family 15 and are distinct from the well‐characterised Snf2 class of ATP‐dependent chromatin remodelling enzymes. AAA‐ATPases typically form hexameric chaperones that utilise the energy from ATP hydrolysis to mediate the folding/unfolding of client proteins 15. The presence of a bromodomain in ATAD2 suggests that its targets include histones and this is supported by protein interaction studies 6, 16. As such, bromodomain AAA‐ATPases represent a novel group of ATP‐dependent histone chaperones.

Orthologs of ATAD2 and ATAD2B have been identified in a range of eukaryotes including yeasts, worms and mammals. Like humans, *Schizosaccharomyces pombe* has two bromodomain AAA‐ATPases designated Abo1 and Abo2. In contrast, *Saccharomyces cerevisiae* has a single ortholog called Yta7, which was identified as a component of a boundary element that restricts the spread of silencing from the *HMR* locus 17. Yta7 is also required for transcriptional induction of histone genes, the *GAL* gene cluster and early meiotic genes 18, 19. Other evidence is suggestive of a global role in the control of nucleosome density. Yta7 exhibits both physical and genetic interactions with ATP‐independent histone chaperones and also core histones. The Yta7 bromodomain interacts with histone tails in an acetylation‐independent manner 16, and the N‐terminal domain also exhibits an affinity for histones *in vitro*, indicating the presence of a second chromatin binding region 16. Deletion of *YTA7* results in histone over‐accumulation and increased nucleosome density within transcribed sequences 19, 20. Based on this, it has been proposed that Yta7 facilitates nucleosome disassembly. However, it is not known whether this chromatin disassembly function is conserved for other bromodomain AAA‐ATPases and the genome‐wide contribution of these proteins is not well understood. To address these issues, we have analysed the fission yeast, *S. pombe*, which is evolutionarily divergent from *S. cerevisiae* and has proved to be an excellent model for the analysis of chromatin function. Contrary to expectation, we find that *S. pombe* cells lacking Abo1, an ortholog of human ATAD2 and *S. cerevisiae* Yta7, experience a global reduction in nucleosome levels in addition to changes in nucleosome organisation. These defects in chromatin structure result in widespread transcriptional de‐repression, loss of heterochromatic silencing and increased chromosome mis‐segregation.

## Results

### Global role for Abo1 in the control of transcription

**Figure 1:**
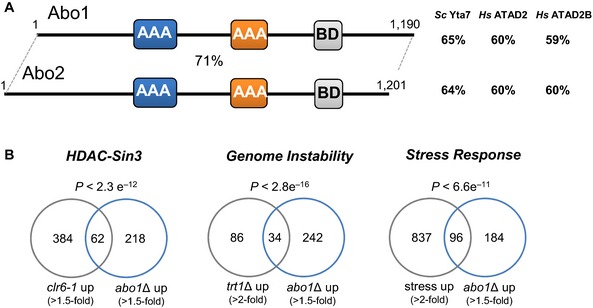
Impact of Abo1 on transcriptome signatures Domain architecture of *Schizosaccharomyces pombe* bromodomain AAA‐ATPases. The D1 and D2 ATPase domains (AAA) are shaded blue and orange, respectively, and the bromodomain (BD) is grey. The percentage similarity to each other and the indicated proteins were determined by FASTA sequence comparison 73 using the scoring matrix BLOSUM50.Venn diagrams showing overlap between genes up‐regulated (≥ 1.5 fold) in *abo1*Δ mutants with genes up‐regulated under the indicated condition, along with the significance of the overlaps (based on hypergeometric distribution).

**Figure EV1:**
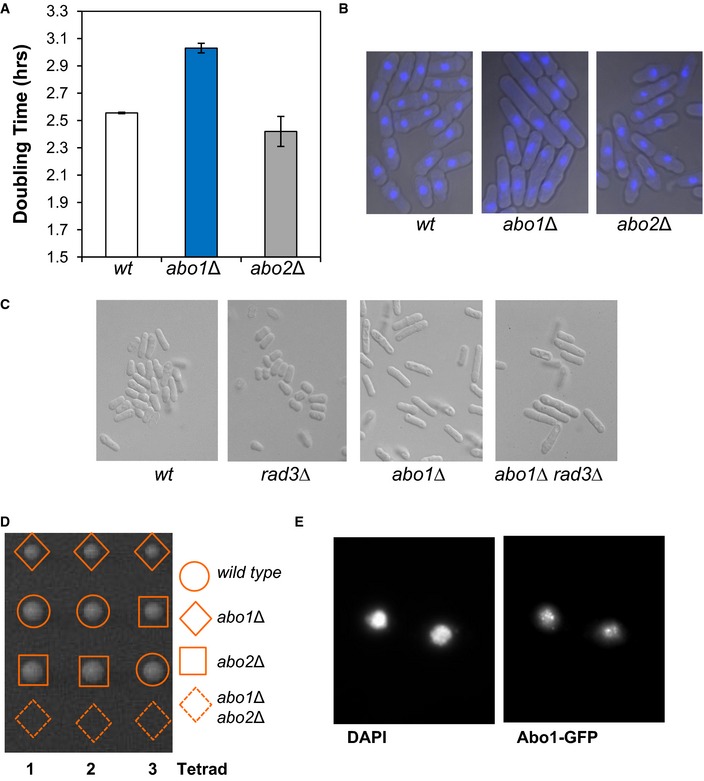
Phenotypes associated with deletion of *abo1* ^+^ Doubling time was estimated by measuring the OD_595_ of the indicated strains grown in YE5S medium at 30°C. Data are the mean of two independent biological repeats, and error bars denote the range of the data.
*abo1*Δ cells have an elongated morphology. Microscopic analysis of DAPI‐stained cells. Data are representative of three biological repeats.The elongated morphology of *abo1*Δ cells is independent of the ATR checkpoint kinase, Rad3. Microscopic analysis of the indicated strain. Images are representative of duplicate experiments.An *abo1*Δ *abo2*Δ double mutant strain is not viable. Tetrad dissection of a genetic cross between *abo1*Δ and *abo2*Δ. Colonies arising from the spores of three asci are shown. Data are representative of three biological repeats.Subcellular localisation of Abo1. Cells expressing Abo1‐GFP were stained with DAPI and visualised using fluorescence microscopy. Data are representative of two biological repeats.

The *S. pombe* genome has two poorly characterised genes, *abo1* ^*+*^ and *abo2* ^*+*^, that encode homologs of human ATAD2/ATAD2B and *S. cerevisiae* Yta7 (Fig 1A). Analysis of a genome‐wide haploid deletion collection has shown that neither *abo1* ^*+*^ nor *abo2* ^*+*^ are essential 21, and therefore, strains carrying null alleles in these genes were subjected to further characterisation. Loss of Abo1 resulted in an increase (~20%) in doubling time and also an elongated cell morphology that is characteristic of a cell cycle delay (Fig EV1A and B). The elongated morphology was retained in the absence of the ATR kinase, Rad3 (Fig EV1C), suggesting that the cell cycle delay is independent of the DNA replication and damage checkpoints. Cells lacking *abo2* ^*+*^ did not display any obvious phenotypes; however, genetic crosses indicated that an *abo1*Δ *abo2*Δ double mutant strain is not viable (Fig EV1D).

We next determined the global transcriptional contributions of Abo1 and Abo2 using microarray analyses. Only a small number of transcripts were reproducibly misregulated in the absence of *abo2* ^*+*^. This is consistent with the phenotypic analysis and data indicating that *abo2* ^*+*^ is expressed at very low levels 22. In contrast, 280 transcripts were reproducibly up‐regulated upon *abo1* ^+^ deletion. These transcripts included mRNAs and also a variety of non‐coding RNAs such as antisense transcripts and snoRNAs. Only 8 transcripts were found to be reproducibly down‐regulated in the *abo1*Δ background, suggesting that Abo1 predominantly functions to suppress transcription, at least in rapidly dividing cells. Consistent with this view, RNAs that were up‐regulated in the *abo1*Δ mutant significantly overlapped (*P* < 2.3 × 10^−12^) with targets of HDAC‐Sin3 co‐repressor complexes 23, 24 (Fig 1B). Further examination of gene expression patterns also revealed a significant overlap (*P* < 2.8 × 10^−16^) between genes that are up‐regulated in the *abo1*Δ mutant and those that are induced in response to genetic instability resulting from telomere crisis 25. It has been established that eukaryotic cells respond to diverse threats to genome integrity by inducing the expression of stress–response genes 26 and accordingly, the *abo1*Δ profile was enriched for core environmental stress–response (CESR) genes 27 (*P* < 6.6 × 10^−11^) (Fig 1B). Overall, the transcriptional signature of the *abo1*Δ mutant is indicative of a stress response resulting from impaired genome integrity.

### Abo1 maintains nucleosome organisation and occupancy at RNAP II transcribed sequences

**Figure EV2:**
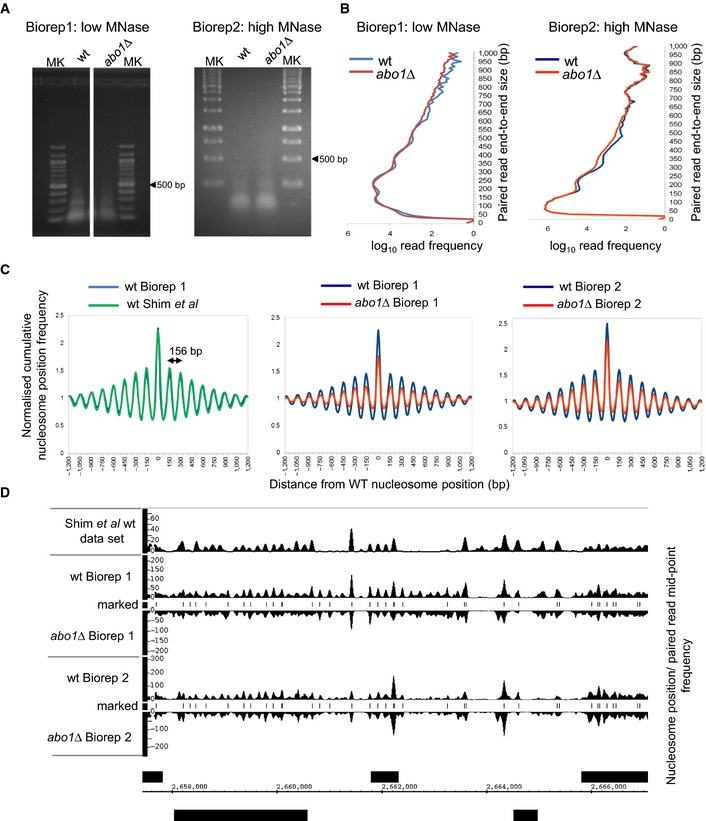
Paired‐end mode chromatin‐seq of *abo1*Δ Ethidium‐stained agarose gel separations of the two DNA pools (Biorep1 and Biorep2) extracted from MNase‐digested *S. pombe* chromatin and used for chromatin sequencing in this study.Frequency distributions of paired‐read end‐to‐end size values after chromatin‐seq of DNAs shown in (A). Note that increased MNase digestion used for Biorep2 samples shifts end‐to‐end size values downwards as expected.Nucleosome positions in wild‐type (Biorep1) cells were defined as the locations of 150 ± 30 bp (nucleosome) size class particle frequency peak summits (frequency value > 25). This simple heuristic procedure identifies 60,658 putative positioned nucleosomes in the *S. pombe* genome. The nucleosome size class particle frequency distributions centred on and surrounding (± 1,200 bp) these positions were then smoothed using an Epanechnikov kernel density estimate (to match that of a previously published data set (Gene Expression Omnibus GSE40451 30), summed and normalised to the average frequency value occurring in the ± 1,200 bp window, for each of the data sets. These cumulative distributions reveal the average nucleosome organisation surrounding positioned nucleosomes in the genome of each cell type. Three pair‐wise comparisons are shown. The nucleosome distribution from wild‐type Biorep1 overlaps with that in a previously published wild‐type data set (Gene Expression Omnibus GSE40451 30), confirming that our nucleosome mapping method yields similar results to those obtained using other technology. The *abo1*∆ mutant nucleosome distributions observed in Bioreps 1 and 2 both show a lower peak height and higher trough depth than the corresponding wild‐type. The wavelength of the peak pattern is shown and is equal to the known *S. pombe* nucleosome repeat length.Genome browser plot of wild‐type nucleosome occupancy data set (Gene Expression Omnibus GSE40451 30) plotted in relation to the 150 ± 30 bp paired‐read mid‐point frequency data obtained in this study (smoothed using an Epanechnikov kernel density estimate). Peak positions match between the two wild‐type data sets, confirming that the 150 ± 30 bp class of paired sequence reads accurately represents nucleosomal species from chromatin. Nucleosome positions in the wild‐type Biorep1 data set defined by our heuristic peak marking procedure are shown as “marked nucleosomes”.

Analysis of the subcellular localisation of Abo1 revealed that it was broadly distributed throughout the nucleus (Fig EV1E), consistent with a global role in the regulation of chromatin. Therefore, the impact of Abo1 on genome‐wide nucleosome organisation was assessed using a chromatin sequencing methodology in which both the position and size of micrococcal nuclease (MNase)‐resistant chromatin species are determined 28. Two bioreps were processed, within which MNase digestion levels for chromatin in *abo1*Δ cells were closely matched to isogenic wild‐type cells (Fig EV2). However, between bioreps, we employed two different levels of MNase digestion (Biorep1 low MNase digestion; Biorep2 high MNase digestion) to control for chromatin regions with enhanced MNase sensitivity 29. Data sets were stratified according to paired‐read end‐to‐end distance into ranges where read pairs of 150 bp (± 20%) derive primarily from mono‐nucleosomes 28. Frequency distributions of the read mid‐points were then mapped to the *S. pombe* genome, and peaks in these distributions were taken to imply the presence of positioned nucleosomes in the cell population (Fig EV2). Bulk positioned nucleosome distributions from wild‐type cells in this study closely matched those determined in a previous study 30, confirming the validity of our technological approach (Fig EV2C). However, the distributions of positioned nucleosomes in *abo1*Δ mutant cells were perturbed relative to wild‐type in both biorep comparisons, suggesting a significant defect in genomic chromatin organisation (Fig EV2C and D).

**Figure 2:**
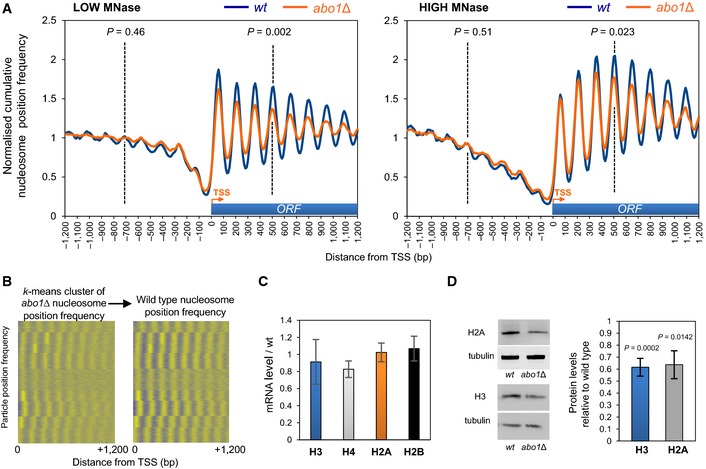
Deletion of *abo1* ^+^ results in the perturbation of nucleosomal organisation at coding sequences Normalised cumulative nucleosome (150 ± 30 bp size class) position frequency profiles for 4,013 *Schizosaccharomyces pombe* genes aligned at the transcription start site (TSS) plotted from low MNase (biorep1) and high MNase (biorep2) data sets. *P‐*values (calculated using a two‐tailed unpaired *t*‐test) for the difference in means between random *n* = 100 subsets of the frequency values at the points indicated by the dotted lines (“−4” nucleosome and “+4” nucleosome) are shown.Nucleosome position frequency values for the coding regions of 4,013 *Schizosaccharomyces pombe* genes were *k*‐means clustered (*k* = 9) using the *abo1*∆ data from biorep1 (low MNase) and displayed with positive values coloured yellow and other values coloured blue (left‐hand panel). The cluster order was then used to display the equivalent wild‐type frequency values in the right‐hand panel.Level of histone gene mRNAs was determined by qRT–PCR. Data are the mean of four independent biological repeats, and error bars are ± SEM. Two‐tailed unpaired *t*‐tests showed no significant differences (*P* > 0.05) between wild‐type and *abo1*Δ cells.Whole‐cell extracts were subjected to Western blotting with histone H3 (Abcam), histone H2A (Abcam) and tubulin antibodies. Examples of the primary data are shown (left) along with a quantification of histone H2A and H3 levels normalised to tubulin (right). Data are the mean of at least three independent repeats, and error bars represent ± SEM. *P‐*values were calculated using a two‐tailed unpaired *t*‐test. Source data are available online for this figure.

Given the change in transcript profile observed in the *abo1*Δ mutant, we focused on the nucleosome architecture surrounding RNA pol II transcription start sites (TSS). Chromatin at the 5ʹ end of eukaryotic genes is typically organised with a nucleosome‐depleted region (NDR) located immediately upstream of the TSS, which is flanked by an ordered nucleosomal array extending into the coding sequence 31, 32. Comparison of average nucleosome positions surrounding TSSs from wild‐type and *abo1*Δ revealed that the NDR and upstream gene regulatory chromatin structure were retained in cells lacking Abo1, as was the average spacing of the reading frame nucleosomal array (Fig 2A). However, the peak amplitude under both MNase digestion conditions was significantly reduced in the mutant background in coding region nucleosomes. Furthermore, there is also a reduction in the depth of the troughs between peaks, suggesting that a re‐distribution of nucleosomes is occurring in the *abo1*Δ mutant cells. We note that under high MNase digestion conditions, the frequency of nucleosome‐sized particles detected in both wild‐type and *abo1*Δ mutant samples dropped in the region directly surrounding the TSS relative to the values observed under low MNase conditions. This result is consistent with the observation that eukaryotic chromatin in this region is labile to nuclease digestion 29.

To examine whether or not a specific set of genes was affected by loss of Abo1, we used *k*‐means clustering to define nine arbitrary nucleosome position peak profiles for *S. pombe* protein‐coding regions in the *abo1*Δ (low MNase digestion) data set (Fig 2B). When this clustering was used to order the nucleosome position peak profiles from the wild‐type data set, no one cluster was found to exhibit a distinct dependence or independence on Abo1. A similar result was observed when this analysis was applied to the high MNase digestion data set (Appendix Fig S1). In both cases, all of the clustered peak profiles identified in the *abo1*Δ mutant resolved more clearly with the wild‐type data set, suggesting that most *S. pombe* protein‐coding gene reading frames exhibit changes to nucleosome organisation to some extent in the absence of Abo1.

**Figure EV3:**
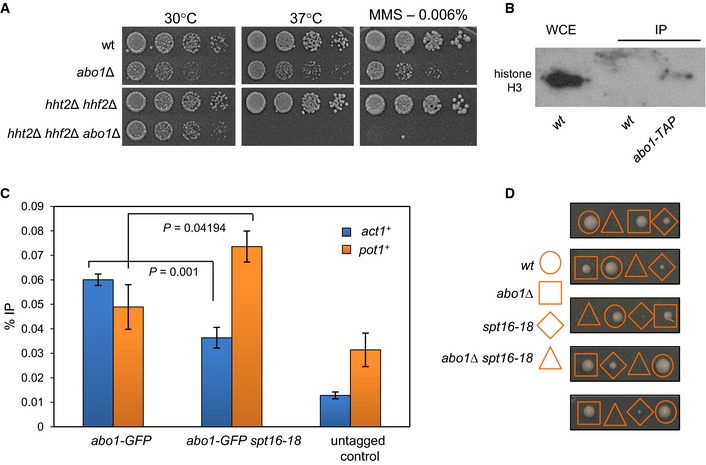
Abo1 physically and genetically interacts with histones and FACT Deletion of the histone H3–H4 gene pair *hht2* ^*+*^ *–hhf2* ^*+*^ exacerbates phenotypes associated with deletion of *abo1* ^*+*^. The indicated strains were grown to mid log phase, subjected to five‐fold serial dilution and spotted onto YES5 plates supplemented as indicated. Plates were incubated for 2 days (37°C) or 3–5 days (30°C). Images are representative of three biological repeats. All strains were present on the same agar plates.Whole‐cell extracts prepared from the indicated strains were partially purified using IgG sepharose and analysed by Western blotting. Data are representative of two biological repeats.ChIP–qPCR analysis was used to detect Abo1‐GFP enrichment at the indicated loci in mid log‐phase cells grown at 30°C. Data are the mean of three independent repeats, and error bars denote ± SEM. *P‐*values were calculated using a two‐tailed unpaired *t*‐test. GFP‐tagged strains exhibit significant enrichment (*P* < 0.05) at both loci relative to the untagged control.Tetrad dissection of a genetic cross between *abo1*Δ and *spt16‐18*. The genotypes of colonies arising from the spores of five asci are shown. Analysis of a total of 28 tetrads from two independent genetic crosses failed to identify a viable double mutant strain.

To determine whether loss of Abo1 affects nucleosome occupancy as well as organisation, we compared histone levels in wild‐type and *abo1*Δ. Although steady‐state histone mRNA levels were not affected by loss of Abo1 when measured using both the microarray data described above and qRT–PCR (Fig 2C), Western analyses revealed significant drops in both histone H3 and H2A protein levels in *abo1*Δ cells (Fig 2D). This indicates that total histone dosage and thus nucleosome occupancy at a global level are lowered in the absence of Abo1. Consistent with this, we found that *abo1*Δ cells are sensitive to DNA‐damaging agents, and deletion of the H3–H4 gene pair *hht2* ^*+*^–*hhf2* ^*+*^ exacerbates this phenotype (Fig EV3A).

### Abo1 associates with the FACT histone chaperone and suppresses cryptic transcription

**Figure 3:**
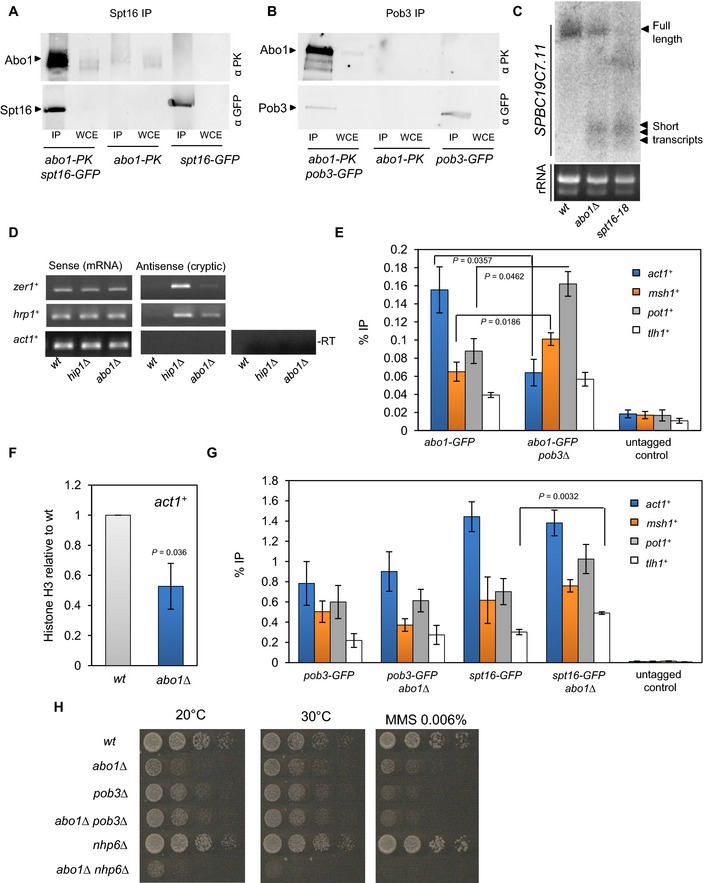
Abo1 associates with FACT and suppresses cryptic transcription A, BWhole‐cell extracts (WCE) were prepared from the indicated strains, immunoprecipitated (IP) with anti‐GFP antibody and subjected to Western blotting with anti‐V5‐Pk (Serotec) and anti‐GFP (Life Technologies) antibodies. Data are representative of three independent biological repeats.CRNA purified from wild‐type, *abo1*∆ and *spt16‐18* cells was analysed by Northern blotting using a probe to the 3′ end of *SPBC19C7.11* (top panel). RNA (5 μg) used for Northern blotting was analysed on an ethidium bromide‐stained 1% TAE agarose gel (bottom panel). Data are representative of two independent biological repeats.DRNA purified from wild‐type and *abo1*∆ cells was analysed by strand‐specific RT–PCR. RNA from *hip1*∆ cells was analysed as a control. One primer, complementary to either the forward or reverse transcripts, was included during the reverse transcription step, and the second primer was then added during PCR amplification. Control reactions omitting the reverse transcription step (−RT) were included to demonstrate the absence of contaminating genomic DNA. Data are representative of two independent biological repeats.EThe indicated strains were subjected to ChIP analysis with anti‐GFP antibodies, and the resulting DNA was analysed by qPCR for the indicated locus. Data are the mean of three independent biological repeats, and error bars represent ± SEM. *P‐*values were calculated using a two‐tailed unpaired *t*‐test. Significant (*P* < 0.05) differences are indicated.FHistone H3 levels at *act1* ^+^ were determined by ChIP–qPCR. Data are the mean of three independent biological repeats, and error bars represent ± SEM. *P‐*value was calculated using a two‐tailed unpaired *t*‐test.GThe indicated strains were subjected to ChIP analysis as described for (E).HLog‐phase cells were subjected to five‐fold serial dilution and spotted onto rich (YE5S) agar or agar supplemented with MMS (0.006%). Plates were incubated for 3–5 days at 30°C or 7 days at 20°C. Images are representative of three independent biological repeats. Source data are available online for this figure.

As Abo1 is required to maintain nucleosome architecture in gene sequences, we hypothesised that it may interact with a histone chaperone that mediates RNAP II transcription‐coupled nucleosome assembly. Indeed, preliminary analysis of Abo1 affinity purifications revealed the presence of subunits of the FACT complex. Therefore, this interaction was probed using co‐immunoprecipitation experiments. Abo1 was found to co‐purify with both Pob3 and Spt16 subunits, confirming an association with FACT (Fig 3A and B). As expected, Abo1 also co‐purified with histone H3 (Fig EV3B).

An increased level of cryptic intragenic transcripts is a hallmark of impaired transcription‐coupled chromatin assembly 1. To determine whether *abo1*Δ mutants experience increased levels of spurious transcription, we examined the *SPBC19C7.11* locus using Northern blotting. As previously reported 33, mutations in *spt16* ^*+*^ resulted in high levels of short abnormal transcripts from this gene and furthermore, similar small transcripts were observed in the *abo1*Δ background (Fig 3C). To further investigate this result, we examined some other genes (*hrp1* ^+^ and *zer1* ^+^) where disruption to RNAP II‐coupled chromatin reassembly is known to result in increased levels of cryptic antisense transcription 24, 34. Strand‐specific RT–PCR confirmed that increased levels of cryptic transcripts were detectable in the absence of *abo1* ^*+*^ (Fig 3D). As such, we conclude that like FACT, Abo1 is required to limit cryptic intragenic transcription.

ChIP analysis revealed that Abo1 was readily detected at ORFs (Fig 3E) ranging from the highly expressed *act1* ^*+*^ gene to the silenced *tlh1* ^+^ gene 22. This is consistent with Abo1 playing a direct role in maintaining nucleosome architecture in transcribed regions, and indeed, histone H3 levels at *act1* ^*+*^ were significantly reduced in the *abo1*∆ background (Fig 3F). As Abo1 co‐immunoprecipitates with both Spt16 and Pob3, we next determined whether FACT influences the recruitment of Abo1 to chromatin or vice versa. Levels of Pob3 and Spt16 at ORFs were not affected by *abo1* ^*+*^ deletion with the exception of *tlh1* ^*+*^ which exhibited significantly increased levels of Spt16 in the *abo1*Δ background (Fig 3G). As discussed further below, we find that Abo1 is required to silence *tlh1* ^*+*^ expression and therefore, the increase in Spt16 levels at this locus may simply reflect increased RNAP II (and thus FACT) recruitment. To determine the influence of FACT upon Abo1 recruitment, we first utilised a *pob3*Δ mutant. Deletion of *pob3* ^*+*^ did not affect Abo1 levels at *tlh1* ^*+*^, but a significant reduction was observed at *act1* ^*+*^. In contrast, at both *msh1* ^*+*^ and *pot1* ^*+*^, Abo1 levels were significantly increased (Fig 3E). Since *spt16* ^*+*^ is an essential gene, it was not possible to determine the impact of its deletion upon Abo1 levels. However, ChIP experiments with the *spt16‐18* allele showed a similar pattern to the *pob3*Δ mutant (Fig EV3C). Thus, it appears that the recruitment of Abo1 to transcription units does not depend upon FACT, but the chromatin distribution of Abo1 is perturbed by mutations in this histone chaperone.

We next examined genetic interactions between *abo1* ^*+*^ and genes encoding subunits of the FACT complex. Analysis of the progeny resulting from genetic crosses between *spt16‐18* and *abo1*∆ strains indicated that the double mutant is not viable indicating that Abo1 is essential when Spt16 function is impaired (Fig EV3D). In contrast, deletion of *abo1* ^*+*^ did not exacerbate the growth or DNA damage sensitivity phenotypes associated with *pob3*∆ (Fig 3H), suggesting that Abo1 functions on the same pathway as Pob3. We also examined the interaction of *abo1*∆ with *nhp6*∆ (*SPAC57A10.09c*) because in *S. cerevisiae,* Nhp6 proteins are loosely associated with Spt16‐Pob3 and promote FACT function *in vivo* 35. Interestingly, we found a strong negative genetic interaction between *abo1*∆ and *nhp6*∆ alleles (Fig 3H).

### Nucleosomes in intergenic regions are disrupted in *abo1*Δ

**Figure EV4:**
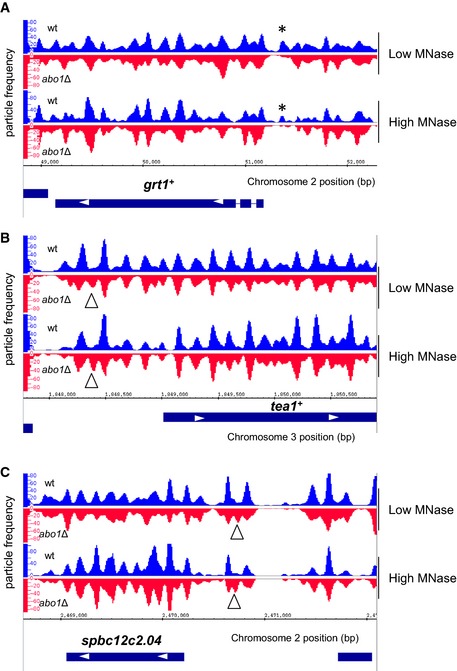
Abo1 is required for the organisation of chromatin in promoters A–CNucleosome (150 ± 30 bp size class) read profile over the indicated chromosomal loci. Data from low MNase (biorep1) are shown in the top panel and data from high MNase (biorep2) in the bottom panel. Missing or additional peaks in the *abo1*∆ background are marked with asterisks and triangles, respectively.

Although average nucleosome positions surrounding TSSs from wild‐type and *abo1*Δ mutant data sets revealed a relatively normal NDR and upstream gene regulatory chromatin structure (Fig 2A), we were also able to detect Abo1‐dependent changes in chromatin structures within intergenic regions. For instance, inspection of the nucleosome peak profiles of *grt1* ^*+*^, which was identified by microarray analysis as an Abo1‐repressed gene, revealed that the −1 nucleosome peak is lost in *abo1*Δ cells (Fig EV4A). We also found examples of promoters (e.g. *tea1* ^*+*^ and *SPBC12C2.04*) where loss of Abo1 resulted in additional mis‐positioned nucleosome peaks (Fig EV4B and C). Thus, Abo1 can also affect the chromatin associated with specific intergenic regions.

### Abo1 is required for heterochromatin

**Figure 4:**
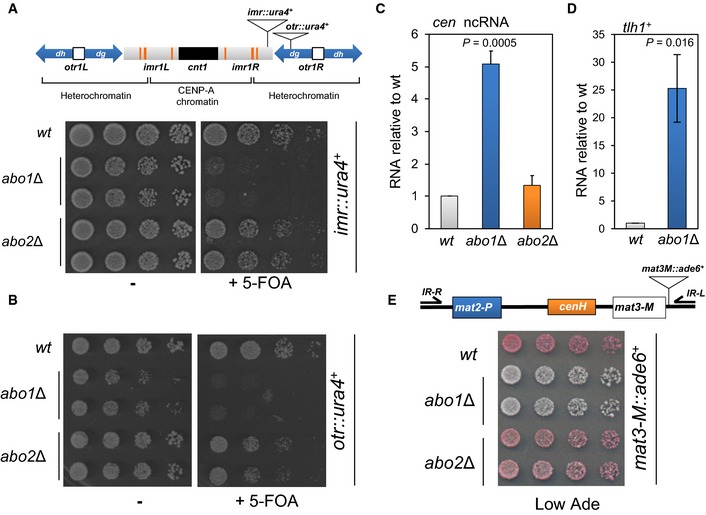
Abo1 is required for heterochromatic silencing The position of *ura4* ^+^ reporter alleles in centromere 1 is shown in the top panel. Strains containing the *imr::ura4* ^+^ allele were grown to log phase in YE5S medium, subjected to five‐fold serial dilutions and spotted onto YE5S agar or YE5S agar supplemented with 5‐FOA (1 mg/ml). Data are representative of three independent biological repeats.Strains carrying *otr::ura4* ^+^ were analysed as described for (A).RNA was purified from the indicated strains and the level of centromeric transcripts determined by qRT–PCR. Data are the mean of three independent repeats, and error bars indicate ± SEM. *P‐*value was calculated using a two‐tailed unpaired *t*‐test.RNA was purified from the indicated strains and subjected to qRT–PCR for the subtelomeric gene, *tlh1* ^+^. Data are the mean of three biological repeats, and error bars indicate ± SEM. *P‐*value was calculated using a two‐tailed unpaired *t*‐test.The position of insertion of *ade6* ^+^ reporter allele in the silent *mat* locus is shown in the top panel. Log‐phase cells containing the *mat3‐M::ade6* ^+^ allele were subjected to five‐fold serial dilution and spotted onto YE5S agar plates lacking adenine (Low Ade). The plates were incubated for 4 days at 30°C. Data are representative of at least three independent biological repeats. Note, microarray analysis indicates that deletion of *abo1* ^+^ does not influence the expression of *ade6* ^+^ (or *ura4* ^+^) when these genes are present at their normal genomic loci.

As FACT is required for heterochromatin in *S. pombe* 36, we determined whether the function of Abo1 also extended to heterochromatic regions such as the pericentromeric *otr* (*dh‐dg*) and outer *imr* repeats. *ura4* ^*+*^ reporter genes located in these repeats are transcriptionally silenced, thereby rendering cells resistant to 5‐FOA. While deletion of *abo2* ^*+*^ did not alter the resistance of either the *otr::ura4* ^*+*^ or *imr::ura4* ^*+*^ reporter strains to 5‐FOA, deletion of *abo1* ^*+*^ resulted in 5‐FOA sensitivity. Therefore, pericentromeric silencing is impaired by loss of Abo1 (Fig 4A and B).

Silencing at pericentromeric regions is dependent upon the RNAi machinery. The *dg‐dh* repeats are bi‐directionally transcribed at low levels by RNAP II, giving rise to long ncRNAs which are processed by Dicer to form siRNAs which promote heterochromatin assembly 37, 38. As the establishment of heterochromatin limits the transcription of repeat sequences, mutations that disrupt its integrity result in the accumulation of long ncRNAs. Consistent with the results of the reporter gene silencing assays, deletion of *abo1* ^*+*^ resulted in a significant (~five‐fold) increase in these ncRNAs relative to wild‐type cells (Fig 4C).

To determine whether Abo1 is also required for heterochromatin at other loci, we examined the *tlh1* ^*+*^ gene which is one of a family of telomere‐linked helicase genes that contain *dg‐dh* repeats similar to those in pericentromeric regions 39. Deletion of *abo1* ^*+*^ resulted in a large (> 20 fold) increase in *tlh1* ^*+*^ expression, indicating that Abo1 is required for subtelomeric heterochromatin silencing (Fig 4D). We also examined the cryptic mating (*mat*) locus as heterochromatin assembly at this site is only partially dependent upon the RNAi pathway due to the existence of a parallel pathway that utilises the Atf1/Pcr1 bZIP transcription factor 40, 41. Wild‐type cells that contain a silenced *mat3‐M::ade6* ^*+*^ reporter form red colonies on adenine‐limiting media (Fig 4E). Silencing was maintained in the *abo2*Δ background, but *abo1*Δ cells formed light pink/white colonies, indicating that silencing had been impaired. Thus, Abo1 is also required for *mat* locus silencing, suggesting that it contributes to heterochromatin structures independently of the RNAi pathway.

**Figure 5:**
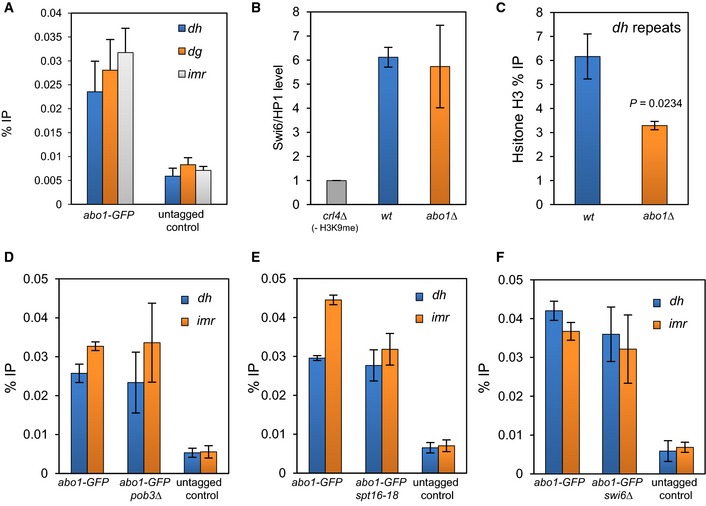
Deletion of *abo1* ^+^ perturbs centromeric heterochromatin ChIP analysis was performed on wild‐type (untagged) and *abo1‐GFP* cells and the resulting DNA analysed by qPCR for centromeric (*dh, dg* and *imr*) repeat sequences. Data are the mean of four independent biological repeats, and error bars represent ± SEM. *P‐*values calculated using a two‐tailed unpaired *t*‐test indicate that all loci are significantly enriched (*P* < 0.05) relative to the untagged control.The indicated strains expressing GFP‐Swi6 were subjected to ChIP analysis. The level of centromeric (*dg*) repeat sequences relative to the euchromatic control locus, *adh1* ^+^, was determined by qPCR and scaled to a *clr4*Δ (‐H3 K9me control) mutant. Data are the mean of two independent ChIP experiments, and error bars represent the range of the data.Histone H3 levels at centromeric (*dh*) repeat sequences were determined by ChIP–qPCR. Data are the mean of three independent ChIP experiments, and error bars are ± SEM. *P‐*values were calculated using a two‐tailed unpaired *t*‐test.ChIP analysis of Abo1‐GFP at centromeric (*dh* and *imr*) repeat sequences regions in wild‐type and *pob3*Δ cells. Data are the mean of three independent experiments, and error bars represent ± SEM. *P‐*values, calculated using a two‐tailed unpaired *t*‐test, indicated no significant difference (*P* > 0.05) for wild‐type and *pob3*Δ cells.ChIP analysis of Abo1‐GFP at centromeric (*dh* and *imr*) repeat sequences in wild‐type and *pob3*Δ cells. Data are the mean of duplicate experiments, and error bars represent the range of the data.ChIP analysis of Abo1‐GFP over the *dh* and *imr* regions in wild‐type and *swi6*Δ cells. Data are the mean of three independent experiments, and error bars represent ± SEM. *P‐*values, calculated using a two‐tailed unpaired *t*‐test, indicated no significant differences (*P* > 0.05) for wild‐type and *swi6*Δ cells.

**Figure EV5:**
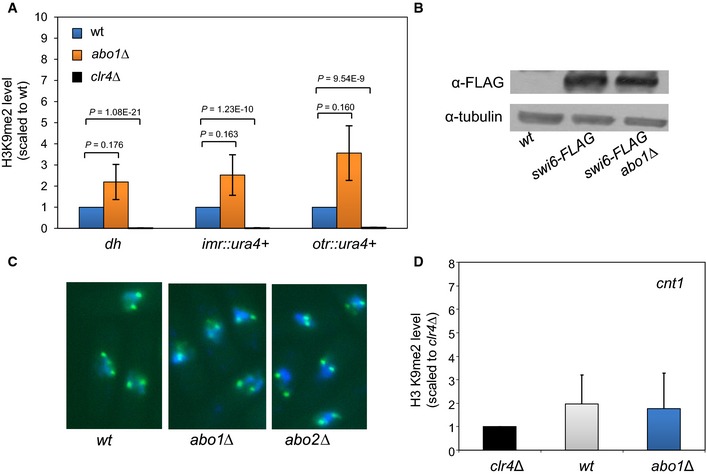
Loss of Abo1 does not reduce H3K9me2 or Swi6/HP1 H3K9me2 levels at *dh* repeats and a centromeric *ura4* ^+^ marker gene were determined by ChIP–qPCR. H3K9me2 enrichment relative to *adh1* ^+^ was determined and levels scaled to wild‐type. Data are the mean of three independent biological replicates, and error bars represent ± SEM. *P‐*values were calculated using a two‐tailed unpaired *t*‐test.Whole‐cell extracts prepared from the indicated strains were analysed by Western blotting with the indicated antibodies. Data are representative of two independent biological repeats.Cell expressing GFP‐Swi6 were stained with DAPI and visualised using fluorescence microscopy. Data are representative of two independent biological repeats.Loss of Abo1 does not result in the inappropriate spread of H3K9me2 into the centromeric central core. ChIP analysis of H3K9me2 levels at central core (*cnt*) sequences of chromosome 1. H3K9me2 enrichment relative to *adh1* ^+^ was determined and levels scaled to the *clr4*Δ (‐H3 K9me2) mutant. Data are the mean of three independent ChIP experiments, and error bars are ± SEM.

To address the mechanism by which Abo1 contributes to heterochromatin, we analysed the transcript levels of 65 genes whose products are involved in its assembly and maintenance (Appendix Fig S2). No marked changes in the expression of these genes were observed in *abo1*Δ cells, suggesting that Abo1 regulates heterochromatin directly. Furthermore, ChIP analysis revealed that Abo1 is enriched at *dh*,* dg* and *imr* repeats (Fig 5A), consistent with a direct contribution to pericentromeric heterochromatin. However, deletion of *abo1* ^*+*^ did not result in a reduction in pericentromeric H3K9me2 (Fig EV5A) or perturbations to the level or localisation of the HP1 homolog Swi6 (Figs 5B and EV5B and C). Furthermore, analysis of H3K9me2 levels associated with central core sequences of centromere 1 (*cnt1*) revealed no difference between wild‐type cells and the *abo1*Δ mutant, suggesting that Abo1 is also not required to prevent the spread of heterochromatin into the CENP‐A containing chromatin in the central core (*cnt*) (Fig EV5D).

Nucleosome position frequency values observed in the MNase‐seq data within the pericentromeric repeat regions were relatively low compared to those found associated with arrays of positioned nucleosomes associated with ORF 5′ regions suggesting a more disorganised nucleosome positioning environment. No consistent changes in nucleosome position were evident between the two MNase‐seq experiments. However, ChIP analysis did reveal a significant reduction in histone H3 levels at centromeric (*dh*) repeats (Fig 5C), indicating that Abo1 is required for normal levels of nucleosome occupancy in pericentromeric heterochromatin.

We next determined whether the recruitment of Abo1 to heterochromatin repeats was dependent upon FACT. Mutations in *spt16* ^+^ and *pob3* ^*+*^ did not influence the level of Abo1 at centromeric repeats, suggesting that its recruitment to heterochromatin is FACT independent (Fig 5D and E). Moreover although Abo1 has previously been show to co‐purify with Swi6/HP1 42, deletion of *swi6* ^*+*^ did not alter the level of Abo1 enrichment at centromeric repeats (Fig 5F). Taken together, our data indicate that Abo1 is recruited to heterochromatin in a FACT‐ and Swi6‐independent manner where it is required to maintain proper nucleosome occupancy.

### Abo1 represses LTR retrotransposons

**Figure 6:**
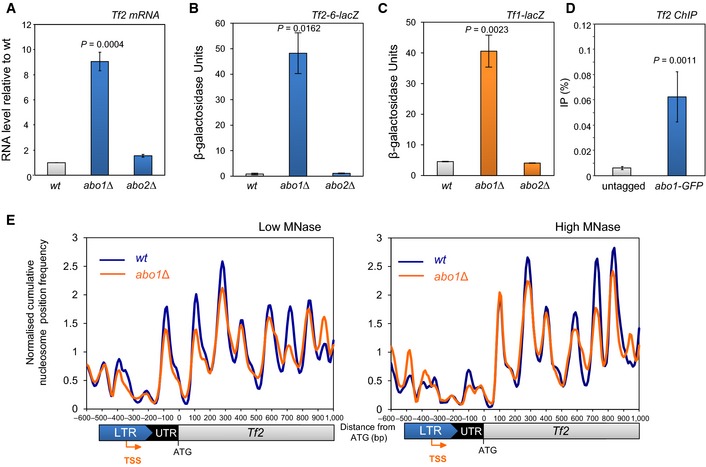
Abo1 represses LTR retrotransposons ARNA was extracted from mid log‐phase cells, and *Tf2* mRNA levels were determined by qRT–PCR, normalised to *act1* ^+^ mRNA and scaled relative to the wild‐type level. Data are the mean of three independent biological repeats, and error bars represent ± SEM.B, CMid log‐phase cells with the indicated integrated *lacZ* reporter were subjected to quantitative β‐galactosidase assays. Data are the mean of three independent biological repeats, and error bars represent ± SEM.DChIP DNA samples from wild‐type (untagged) and *abo1‐GFP* cells were analysed by qPCR for *Tf2* LTR. Data are the mean of four independent biological repeats, and error bars represent ± SEM.ENormalised cumulative nucleosome (150 ± 30 bp) position frequency profiles for *Tf2* LTR retrotransposons aligned at the ATG plotted from low MNase (biorep1) and high MNase (biorep2) data sets.Data information: *P‐*values were calculated using a two‐tailed unpaired *t*‐test.

The microarray analyses suggested that Abo1 also represses the expression of *Tf2* LTR retrotransposons. Although these retroelements are silenced, they are not enriched with H3K9me 43 and are instead subjected to a distinct form of silencing 34, 44, 45, 46, 47, 48. Quantitative RT–PCR and reporter gene analyses confirmed that the suppression of *Tf2* mRNA levels is also dependent upon Abo1 (Fig 6A and B). The 13 *Tf2* elements are the only full‐length retrotransposons in the genome of the *S. pombe* reference strain, although a related element, *Tf1,* has been present during its evolution and intact *Tf1* elements can be isolated from other wild‐type *S. pombe* strains 49. To address the regulation of *Tf1* elements, we constructed a strain with an integrated *Tf1‐lacZ* reporter and determined that this “extinct” retrotransposon is also silenced in an Abo1‐dependent manner (Fig 6C).

ChIP analysis confirmed that Abo1 is associated with *Tf2* LTR retrotransposons (Fig 6D) and so we determined the impact of Abo1 on the MNase‐seq profiles associated with these elements (Fig 6E). The peak downstream of the TSS was found to be highly sensitive to MNase digestion, suggesting that this nucleosome is labile. In the *abo1*∆ background, changes in the amplitude of specific peaks were observed in both data sets consistent with a perturbation to local nucleosome architecture at *Tf2* LTR retrotransposons.

### Abo1 is required for accurate chromosome segregation

**Figure 7:**
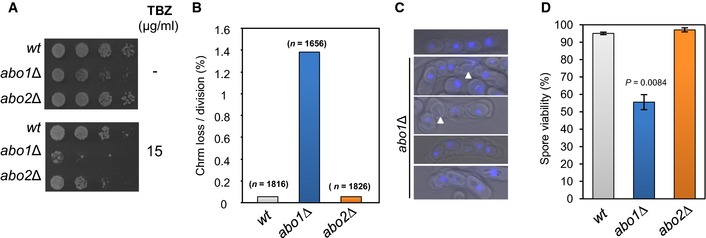
Abo1 is necessary for accurate chromosome segregation Log‐phase cells were subjected to five‐fold serial dilution and spotted onto rich (YE5S) agar or agar supplemented with TBZ (15 μg/ml) and incubated for 3–5 days at 30°C. Data are representative of three independent biological repeats.The frequency of *Ch16* minichromosome loss was assayed by plating cells onto adenine‐limiting agar and determining the frequency of half‐sectored (red‐white) colonies. At least two independently derived strain isolates were used for each genotype, and the total number of colonies counted for each genotype is indicated in parentheses.Heterothallic (*h* ^*90*^) strains were cultured on nitrogen‐limiting (EMMG) medium for 3 days at 25°C to induce mating and meiosis. The resulting asci were stained with DAPI and visualised by fluorescence microscopy. Spores lacking a DAPI signal are indicated by arrowheads. Data are representative of three biological repeats.Spore viability was measured by dissecting spores onto YE5S agar followed by incubation for 4–5 days at 30°C. Data are the mean of three independent biological repeats, and error bars represent ± SEM. *P‐*value was calculated using a two‐tailed unpaired *t*‐test.

Our findings predicted that deletion of *abo1* ^*+*^ will lead to dysfunctional centromeres. Therefore, we analysed the sensitivity of *abo1*Δ cells to thiabendazole (TBZ), a drug which depolymerises microtubules and impairs mitotic spindle function. In common with other mutants that have defective centromeres, *abo1*Δ cells were sensitive to TBZ (Fig 7A). To further investigate the requirement of Abo1 in chromosome segregation, we analysed the mitotic stability of a 0.5‐Mbp linear mini‐chromosome, Ch16 50. Deletion of *abo1* ^*+*^ resulted in a ~25‐fold increase in the loss rate of Ch16*,* confirming that it is required for accurate mitotic chromosome segregation (Fig 7B).

To analyse the role of Abo1 in meiosis, the *abo1*Δ allele was introduced into a homothallic (*h* ^*90*^) background. Then mating and meiosis were induced by starving cells for nitrogen. The *h* ^*90*^ *abo1*Δ cells also showed no obvious defect in their ability to conjugate, but they frequently produced asci with an aberrant number of spores, a proportion of which failed to display a DAPI signal (Fig 7C). To quantify this defect, spore viability was determined and was found to be significantly reduced (~50%) in *abo1*Δ (Fig 7D). Therefore, the changes in chromatin structure that result from loss of Abo1 function lead to major defects in both the mitotic and meiotic cell cycles.

## Discussion

Here, we report the global chromatin functions of a bromodomain AAA‐ATPase. Cells lacking *abo1* ^*+*^ have perturbed nucleosome organisation in transcribed regions and a corresponding increase in cryptic transcription which are features of mutants that are defective in transcription‐coupled chromatin assembly. Consistent with this, Abo1 interacts with histone H3 and the essential histone chaperone FACT, and furthermore, the level of Abo1 at some genes is altered by mutations in FACT subunits. However, the impact of Abo1 upon nucleosome structure was not restricted to transcribed gene sequences, and so like FACT, it may also promote the proper re‐establishment of chromatin in other contexts such as replication.

Our finding that Abo1 promotes nucleosome occupancy was unexpected given previous analysis of *S. cerevisiae,* which indicates that Yta7 facilitates nucleosome removal 19, 20. Nucleosome density within genes downstream of the +1 nucleosome increases in the absence of Yta7, and furthermore, the phenotypes associated with its loss can be suppressed by reducing histone H3–H4 dosage. In contrast, there is an overall reduction in histone content in cells lacking Abo1 and we also find that reducing histone H3–H4 gene dosage exacerbates the temperature and DNA damage‐sensitive phenotypes of *abo1*Δ cells. At first glance, it is difficult to reconcile the disparate phenotypes that result from loss of bromodomain AAA‐ATPases in budding and fission yeast. However, it is becoming increasingly evident that ATP‐independent histone chaperones that mediate nucleosome assembly are also capable of promoting the reverse reaction of disassembly 51. Likewise, bromodomain AAA‐ATPases may participate in both assembly and disassembly reactions in a context‐dependent manner. It is also possible that Abo2 mediates nucleosome removal; however, Abo2 is expressed only at very low levels. Indeed, in a recent proteomic analysis, Abo2 was not detectable, while Abo1 is relatively abundant (~1,600 molecules per cell) 22. Furthermore, our microarray analysis indicates that loss of Abo1 does not lead to an increase in *abo2* ^*+*^ expression. Therefore, it is highly unlikely that the reduced nucleosome levels in *abo1*Δ result from the up‐regulation of Abo2.

Chromatin‐seq analysis revealed that Abo1 impacts upon nucleosome positioning in coding sequences. Indeed, the peak profiles suggest that in *abo1*Δ cells, many nucleosomes become “fuzzy” and shift from occupation of a single favoured position. Regular nucleosome spacing over genes sequences is controlled by ISWI and CHD chromatin remodelling complexes 30, 52, 53. In *S. pombe* (which lacks ISWI complexes), loss of the CHD remodelers Hrp1 and Hrp3 abolishes regularly spaced nucleosomal arrays over transcribed sequences 30. Given that Abo1 is an AAA‐ATPase, it is unlikely that it has any direct nucleosome spacing/sliding activity. Furthermore, nucleosomal arrays are not completely lost in *abo1*Δ, as is the case in *hrp3*Δ *hrp1*Δ mutants, suggesting that Abo1 is not necessary for the function of these CHD remodelers. However, we cannot exclude the possibility that Abo1 may promote proper localisation of these or other chromatin modifying complexes. In this context, it is interesting that human ATAD2 controls the chromatin association of the MLL histone methyltransferase complex 6.

The phenotypes of the *abo1*Δ strain are reminiscent of *S. cerevisiae* mutants which lack the HMGB proteins, Nhp6a and Nhp6b. These mutants experience a substantial drop in nucleosome numbers and also a proportion of the remaining nucleosome positions become fuzzy 54. On this basis, it has been argued that Nhp6 proteins provide a chaperone‐like activity for nucleosome assembly 54. An alternative interpretation is that Nhp6 proteins function to increase nucleosome stability 55. Equally, the *abo1*Δ phenotypes are open to both interpretations. While these modes of action are not mutually exclusive, a prediction of the stabilisation model is that nucleosomes would become increasingly labile in the absence of Abo1 and that rates of H2A–H2B dimer exchange may increase. It is therefore noteworthy that photobleaching experiments suggest that H2A turnover is increased by knockdown of human ATAD2 12. The impact of *S. pombe* Nhp6 on global nucleosome occupancy and organisation has not yet been determined. However, that the loss of Nhp6 enhances the growth and DNA damage phenotypes associated with Abo1 inactivation suggests that they contribute to the maintenance of chromatin via distinct pathways.

*Saccharomyces cerevisiae* Yta7 was originally identified as a component of a tRNA gene‐containing barrier element that prevents the inappropriate spread of silencing from *HMR* 17. While the *S. pombe* barriers that prevent the invasion of heterochromatin into the central core of centromere 1 are also dependent upon tRNA genes 56, they apparently do not require Abo1. Instead, our finding that Abo1 is required for silencing provides the first demonstration that bromodomain AAA‐ATPases can contribute to the function of heterochromatin. Heterochromatin is characterised by the association of the HP1 proteins which form a platform for the assembly of an array of additional silencing factors (such as histone chaperones, histone deacetylases and ATP‐dependent chromatin remodelling enzymes). Although Abo1 also appears to function as one of these silencing factors and is known to interact with Swi6/HP1 42, its recruitment to heterochromatin is not dependent upon Swi6/HP1. Furthermore, the dysfunction of centromeric heterochromatin in the *abo1*Δ background does apparently result from reduced levels of Swi6/HP1. However, ChIP analysis revealed that loss of Abo1 leads to changes in nucleosome occupancy over centromeric repeats. Furthermore, recent studies indicate that relatively subtle changes to either nucleosome positioning or occupancy in centromeric heterochromatin can be sufficient to alleviate silencing and drive chromosome mis‐segregation events 30, 57.

Histone post‐translational modification and ATP‐dependent chromatin remodelling represent key mechanisms for modulating chromatin structure and function. However, controlling whether or not a particular DNA sequence is assembled into a nucleosome represents an even more profound mechanism for influencing chromatin structure. Thus, the regulation of nucleosome occupancy may be considered a primary layer of chromatin control. In this respect, it is now apparent that nucleosome number within cells is not fixed 54. For instance, both ageing yeast and mammalian cells exhibit reduced histone and nucleosome content 58, 59. Here, we demonstrate that a bromodomain AAA‐ATPase is required for global nucleosome occupancy, indicating that these factors play a key role in regulation of this primary layer of chromatin control.

## Materials and Methods

### *Schizosaccharomyces pombe* strains

Routine culture and genetic manipulation were performed as previously described 60. The strains used in this study are listed in Appendix Table S1. Strains carrying *kanMX* replacements of ORFs *SPAC31G5.19* and *SPBP22H7.05c* were purchased from Bioneer, genotyped by PCR analysis and backcrossed with the appropriate strains. A strain expressing Pk‐tagged Abo1 was constructed by cloning a PCR fragment (amplified with primers CAGTTCGGATCCTTGTAGTGCATTAATCATAAACTC and GTTCACCTGCAGAATTAAGGCACGGAAAGCTTC) into the *Pst*I and *Bam*HI sites of pRip42‐Pk 61. The resulting plasmid was digested with *Bcl*I and transformed into *S. pombe*. A strain expressing Abo1 tagged with GFP was constructed using the approach described in 62. Phenotypic analysis demonstrated that epitope‐tagged versions of Abo1 are functional (Appendix Fig S3). A strain with a *Tf1‐lacZ* reporter integrated into chromosome II (base pairs 1,877,855–1,878,381) was constructed by PCR amplifying a DNA fragment using template DNA derived from wild strain NCYC132 and with primers (GCTAAGCTGCAGTGTCAGCAATACTACACTACGCTA) and (GGAT GCCTGCAGGCTGTTCAGTTGAATATCTATCGG). The fragment was cleaved with *Pst*I and cloned into the *Pst*I site of pSPI356ChrmIICDAB 34. The resulting plasmid was linearised with *Bam*HI and transformed into the appropriate strain *S. pombe* cells. Quantitative β‐galactosidase assays were performed as previously described 63.

### Microarrays

RNA was extracted using hot phenol and purified over RNase easy columns (Qiagen) as previously described 64. A 15‐μg aliquot of total RNA was prepared for microarray analysis using the Superscript Plus Direct labelling kit (Invitrogen, Life Technologies), in two biological repeats with dye swaps. Paired samples were hybridised over two 44K Custom microarrays (Agilent) overnight at 65°C, scanned using GenePix 4000B scanner (Axon Instruments) and analysed using Genepix Pro 6.0 software(Axon Instruments). In house normalisation, scripts were applied (to gpr files) and the data uploaded into Genespring 7.3.1 software for analysis. Microarray data can be accessed at ArrayExpress accession E‐MTAB‐3455.

### Histone levels

Approximately 4 × 10^7^ cells were harvested following the addition of trichloroacetic acid (TCA) to a final concentration of 10%. Cells were resuspended in 200 μl 10% TCA and then disrupted using a beadbeater with 0.75 ml of glass beads using two pulses of 15 s with 1 min on ice in between. A 500‐μl aliquot of 10% TCA was added, and the lysate was recovered from the beads which was then clarified by spinning at 15,000 × *g* in a microcentrifuge. The resulting pellet was washed three times in acetone, dried and resuspended in 30 μl 100 mM Tris–HCl (pH 8.0), 1% w/v SDS and 1 mM EDTA. Samples were analysed on SDS–PAGE gels and subjected to Western blotting using anti‐histone H3 (Abcam ab1791), anti‐histone H2A (Abcam ab13923) and anti‐tubulin (TAT‐1) antibodies. Western blots were visualised using a Typhoon FLA9500 (GE Healthcare) and band intensities quantified using ImageQuant.

### RT–PCR and Northern analyses

RNA was purified as described for microarray analysis and subjected to RT–PCR using a One Step RT‐PCR kit (Qiagen). For strand‐specific RT–PCR, one primer complementary to the sense or antisense transcript was added during first‐strand cDNA synthesis, while the second primer was added prior to the PCR amplification steps. cDNA for quantitative (real‐time) RT–PCR was made using a Superscript II kit (Invitrogen). Real‐time PCRs were performed using a LightCycler 2.0 PCR system (Roche) and SYBR Green mix (Molecular Probes) using the appropriate primers. Reactions were normalised using primers specific to *act1* ^*+*^. Northern analysis was performed as previously described 65.

### MNase digestion of chromatin

Cells (100 ml) were grown to OD_595_ = 0.75–8.0 in YE5S at 30°C, cross‐linked for 20 min at 30°C using 1% formaldehyde and quenched by the addition of glycine to 125 mM. Cells were washed once in CES buffer (50 mM citric acid/50 mM Na_2_HPO_4_ [pH 5.6], 40 mM EDTA [pH 8.0], 1.2 M sorbitol and 10 mM β‐mercaptoethanol) and resuspended in 500 μl of CES buffer with 0.5 mg Zymolase 100‐T. Cells were spheroplasted at 30°C for up to 1 h and then washed twice with ice‐cold 1.2 M sorbitol. Spheroplasts were then resuspended in 800 μl NP‐S buffer (1.2 M sorbitol, 10 mM CaCl_2_, 100 mM NaCl, 1 mM EDTA pH 8.0, 14 mM β‐mercaptoethanol, 50 mM Tris pH 8.0, 0.075% NP‐40, 5 mM spermidine, 0.1 mM PMSF, 1% Sigma protease inhibitors cocktail [Sigma P8215]). Spheroplasts were then divided into four 200 μl aliquots, and each aliquot was mixed with 300 μl of NP‐S buffer. Three aliquots were digested with between 75 and 187.5 units of MNase (USB) for 10 min at 37°C. The fourth was retained as an undigested control. MNase digestion was terminated by adding EDTA [pH 8.0] to a final concentration of 50 mM and SDS to 0.2%. Reactions were incubated at 65°C overnight with 0.2 mg/ml proteinase K and 10 μg RNAse. DNA was purified by extracting twice with phenol:chloroform followed by ethanol precipitation (0.1 volumes of 3 M sodium acetate followed by two volumes of ethanol). Pellets were washed in 70% ethanol and resuspended in water containing 10 μg/ml RNase and incubated at 37°C for 30 min. Triplicate digests were pooled and treated with 100 U unmodified T4 polynucleotide kinase (NEB) for 30 min at 37°C to remove 3′‐phosphate groups left by MNase. DNA was extracted once more with phenol:chloroform, re‐precipitated with sodium acetate and propan‐2‐ol, washed with 80% ethanol, dried and re‐suspended in TE (pH 7.5).

### Chromatin‐seq

DNA fragments were end repaired, 3′‐adenylated and ligated to indexed adapters without size selection using Nextflex reagents (Newmarket Scientific, UK). Libraries were amplified with eight cycles PCR using Kapa HiFi PCR master mix (Anachem), primers removed with GeneRead size selection protocol (QIAgen) before quantification by Bioanalyser DNA 7500 assay. Libraries were pooled, denatured and diluted to 6 nM before clustering in a single lane of a high‐output Illumina flow cell. Sequencing (100 nt) was undertaken on a HiSeq 2500 using TruSeq SBS v3 reagents (Illumina).

### Bioinformatics

Paired reads were aligned to the ASM294v1.17 reference genome using Bowtie 0.12.7 66 with command line flags: ‐n 0 –trim3 64 –maxins 5000 –fr ‐k 1 –sam. Aligned read pairs were sorted according to chromosome and then into a range of size classes based on the SAM format ISIZE value (difference between 5′ end of the mate read and the 5′ end of the first mapped read) plus or minus 20%. Mono‐nucleosome‐sized reads are, therefore, represented as 150 ± 30 bp. To define the genomic position of MNase‐resistant chromatin entities, we mapped the mid‐point position of the read pairs in a particular size class. Frequency distributions of the mid‐point positions were then calculated using 10‐bp bins. To mark positions of peak maxima, and for *k*‐means clustering, the frequency distributions were lightly smoothed by taking a 3‐bin moving average. For all other analyses and displays, the frequency distributions were smoothed using an Epanechnikov kernel density estimate (using the Perl module Statistics::Kernel Estimation‐0.05 with bandwidth = 30) to match the profile of the previously published *S. pombe* nucleosome position data set (Gene Expression Omnibus GSE40451 30). All frequency distributions were output in the zero‐referenced, chromosome base, three‐column.sgr format (chromosome number, feature/bin position, mid‐point frequency value) for rendering with the Integrated Genome Browser 67 and for further processing. Average cumulative chromatin particle position frequency distributions at, and surrounding, genomic features were calculated using the script SiteWriterCFD as described previously 28, 68, with values for each bin normalised to the average cumulative frequency value obtained for all bins within the feature window. Protein‐coding gene transcription start site (TSS) positions were taken from the previously published data set 32. Nucleosome positions in the WT biorep1 data set were defined using a simple heuristic peak summit marking process (script PeakMarker_lite; read frequency threshold set to 25) (Script EV1). Clustering was performed with Cluster 3 using *k*‐means and the Euclidean distance similarity metric 69. Clusters were displayed using JavaTreeView 1.1.6 with values plotted as log_2_; centre = 3.0; and contrast value = 10. Wild‐type DNA sequence data (low MNase digestion) can be found in the NCBI Sequence Read Archive (SRA): accession number SRS712792. Other MNase sequence data can be accessed via Gene Expression Omnibus GSE67410.

### ChIP analysis

For Swi6 ChIP, strains were grown in PMG complete media to exponential growth phase at 25°C. Cells were then fixed with 3% formaldehyde for 18 min at room temperature. Chromatin immunoprecipitation was performed as previously described 70. In short, fixed cells were suspended in 400 μl ChIP lysis buffer (50 mM HEPES [pH 7.5], 140 mM NaCl, 1 mM EDTA, 0.1% DOC, 1% Triton X‐100) and disrupted by beadbeating (2 × 1 min). Chromatin was then sheared by probe sonication (3 × 20 s) and immunoprecipitated with anti‐GFP (Life Technologies). Cross‐linking was reversed by overnight incubation with TES followed by proteinase K digestion. After DNA purification, samples were subject to real‐time qPCR. Abo1‐GFP, Spt16‐GFP, Pob3‐GFP, histone H3 and H3K9me2 ChIP were performed as above but with the following modifications; cells were grown at 30°C in YE5S and were fixed by the addition of formaldehyde to a final concentration of 1% for 15 min at room temperature; chromatin was sheared using a Diagenode Bioruptor using approximately 30 cycles (30 s ON/OFF). Chromatin was immunoprecipitated with anti‐GFP (Life Technologies A11122), anti‐H3 (Abcam ab1791) or anti‐H3K9me2 (Abcam ab1220). Immunoprecipitated DNA was recovered as described previously 71.

### Co‐immunoprecipitations

Whole‐cell extracts, prepared as previously described 65, were pre‐cleared with 40 μl Sepharose A beads for 1–2 h at 4°C on rotating wheel. The resulting supernatant was transferred to a fresh microcentrifuge tube and incubated with 40 μl Sepharose A beads and 1.5 μl anti‐GFP (Life Technologies) at 4°C overnight on a rotating wheel. Sepharose A beads were harvested by centrifugation and washed 3–6 times in buffer (50 mM Tris–HCl [pH 7.5], 150 mM NaCl, 10 mM imidazole, 0.5% [v/v] NP‐40 [IGEPAL], 1 mM PMSF, 54 mM NaF, 5 μM NaVO_4_, 1 μl/ml aprotinin, 5 μg/ml pepstatin A and 5 μg/ml leupeptin) before being resuspended in 40 μl Laemmli buffer (0.1% β‐mercaptoethanol, 0.0005% bromophenol blue, 10% glycerol, 2% [w/v] SDS and 63 mM Tris–Hcl [pH 6.8]). Samples were then analysed on 9% SDS–polyacrylamide gels followed by Western blotting.

### Microscopy

Cells were pelleted in a microcentrifuge at 3,000 × *g* for 2 min before pellets were resuspended in ~20 μl of medium. Microscope slides were coated in poly lysine and allowed to dry before the addition of 5 μl of the cell suspension. Cells were allowed to dry, and 5 μl DAPI containing mounting medium (Vectashield^®^ with DAPI [Vector laboratories]) was added. Images were obtained using Zeiss Axiovert 200M with 100× oil immersion lens and Axiovision software.

### Statistics

*P*‐values were calculated using two‐tailed unpaired *t*‐tests, and the statistical significance is shown in the relevant Figure. *P*‐values for the overlaps in microarray gene lists were calculated in Excel and are based on hypergeometric distribution using all coding genes as the background population size. For comparison of nucleosome position frequency values at −4 and +4 nucleosomes surrounding protein‐coding gene TSSs, the data were subsampled into 29 random 100 TSSs subsets to avoid the tendency of *P*‐values to become inaccurate with large total *n* 72. Normality and variance were checked using the plot command in R 3.2.2, and *P*‐values were calculated using Perl module Statistics::DependantTTest‐0.03.

## Author contributions

HEM, CG, LS, AJW, KMM, KP, SC, NAK, SKW and KMC performed experiments and analysed data. JB, JFP, RCA, NAK and SKW designed experiments and analysed data. SKW and NAK wrote the manuscript with input from all authors.

## Conflict of interest

The authors declare that they have no conflict of interest.

## Supporting information



Appendix

Expanded View Figures PDF

Script EV1

Review Process File

Source Data for Figure 2

Source Data for Figure 3
