# Supplementary material for: Abo1, a conserved bromodomain AAA‐ATPase, maintains global nucleosome occupancy and organisation
Source: EMBO Rep. 2015 Nov 18;17(1):79–93. doi: 10.15252/embr.201540476 (PMC4718406; doi:10.15252/embr.201540476)
Supplement: Supplementary file 1 — Appendix [file EMBR-17-079-s001.pdf]

APPENDIX ITEMS

Table of Contents

**Page(s)**	**Item**
2	**Appendix Figure S1.** *k*-means clustering of nucleosome position frequency values for *S. pombe* protein coding genes (High MNase dataset).
3	**Appendix Figure S2.** Abo1 does not influence the expression of heterochromatin genes.
4	**Appendix Figure S3.** Phenotypic analysis of *abo1-Pk* and *abo1-GFP* strains
5-7	**Appendix Table S1.** Strains used in this study.

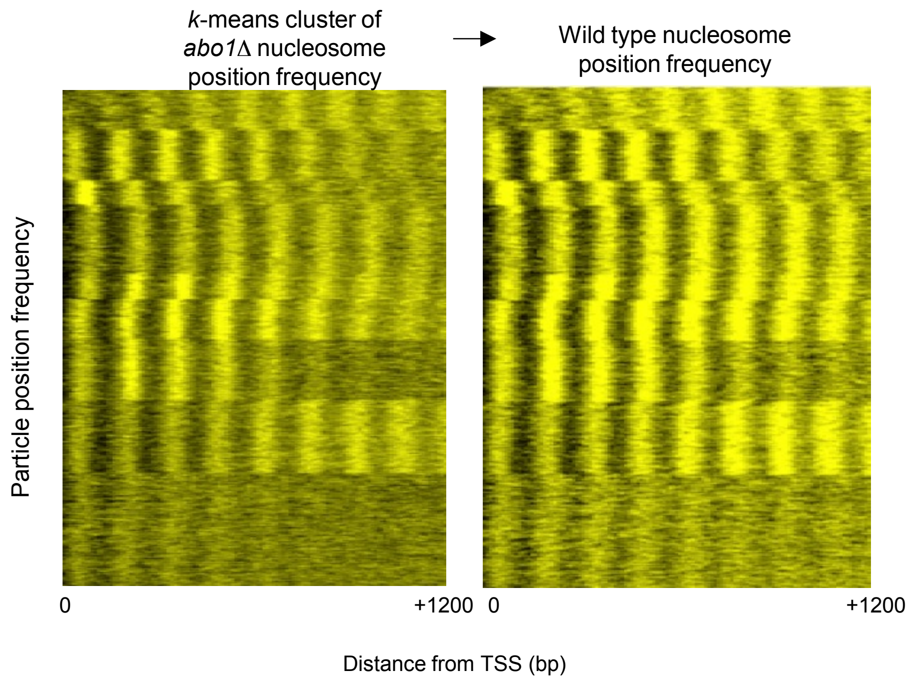

Appendix Figure S1. Nucleosome position frequency values for the coding regions of 4013 *S. pombe* genes were *k*-means clustered (*k* = 9) using the *abo1*Δ data from the high MNase digestion data set and displayed with positive values coloured yellow and other values coloured black (left-hand panel). The cluster order was then used to display the equivalent wild type frequency values (right-hand panel). As observed in Figure 2B, all clustered peak profiles identified in the *abo1*Δ mutant resolved more clearly with the wild type dataset. Note that the order of the clusters displayed here is different to those defined for the low MNase dataset in Figure 2B.

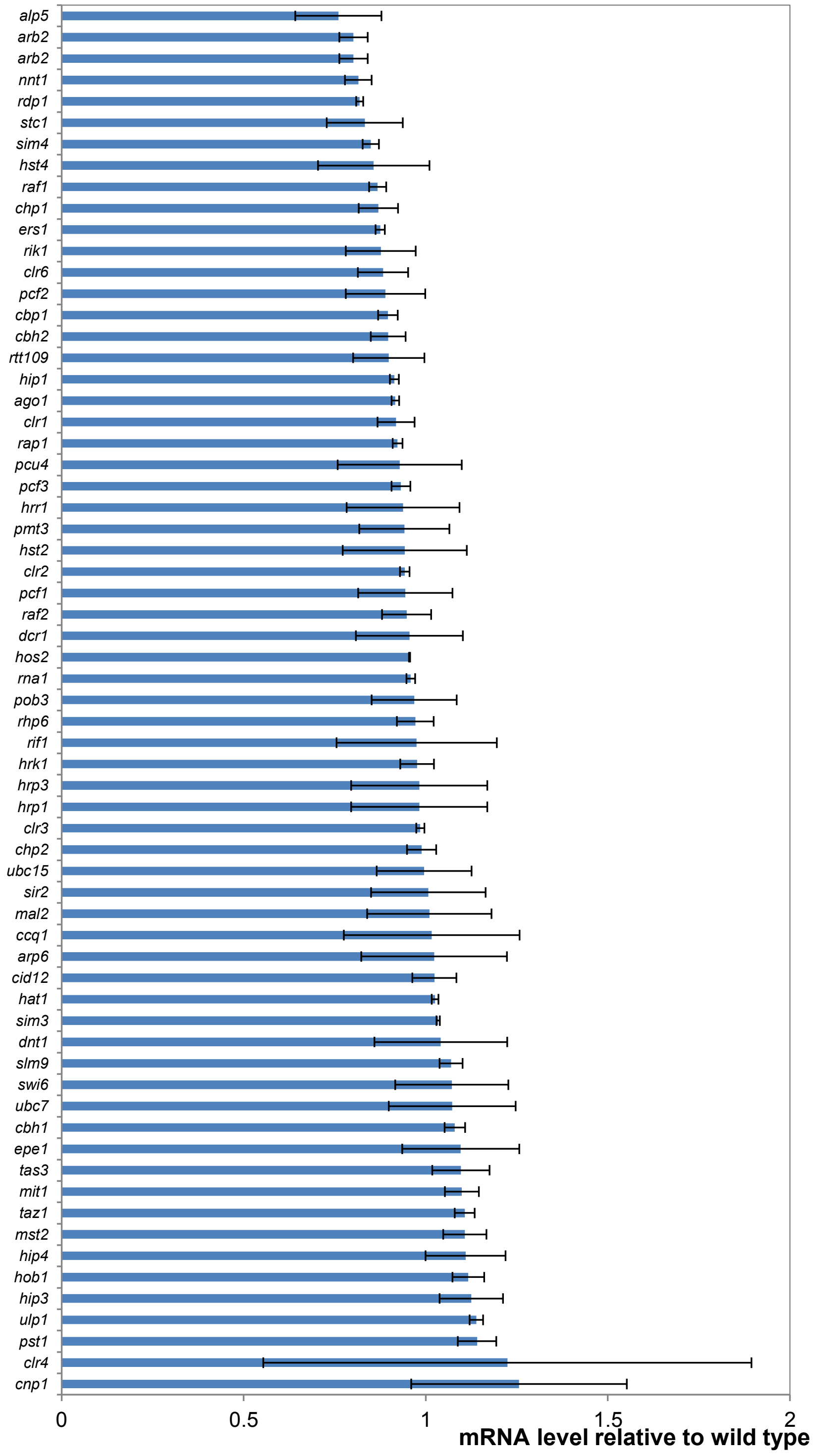

Appendix Figure S2. Abo1 does not influence the expression of heterochromatin genes.

The impact of *abo1*$^{+}$ deletion on the mRNA level of 65 genes whose products contribute to heterochromatin was determined by microarray analysis. RNA level is shown relative to wild type. Means were calculated from duplicate experiments. Error bars indicate the range of the data.

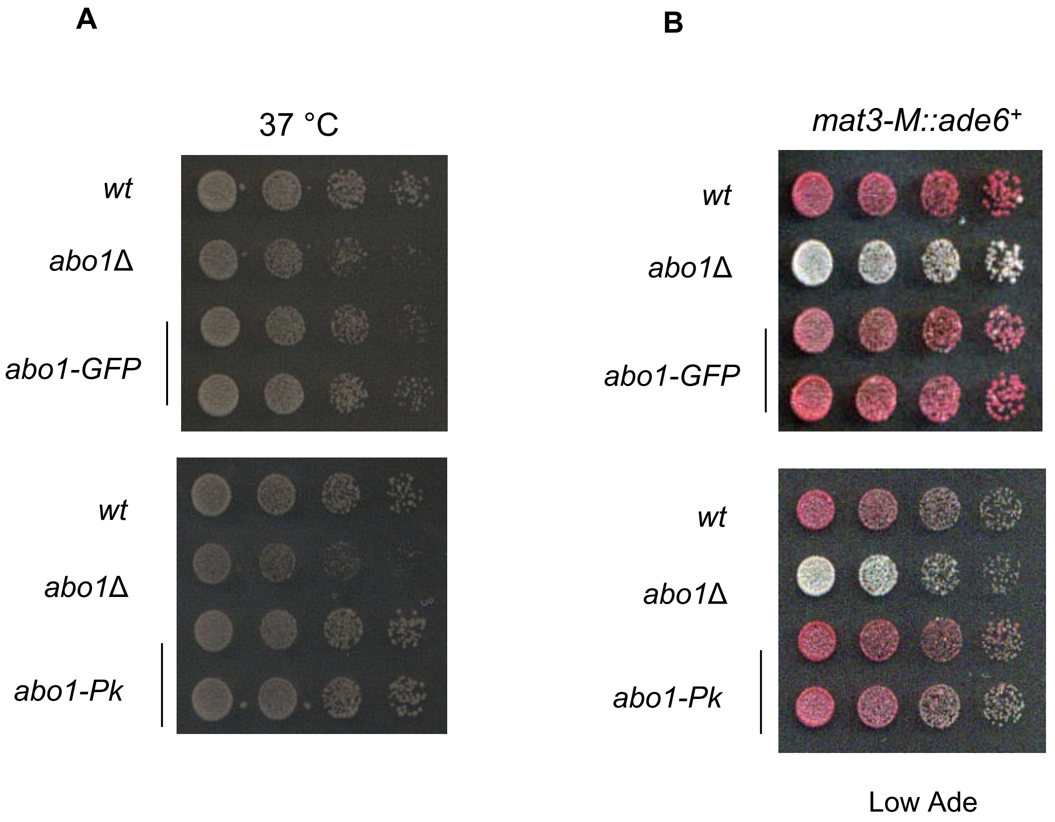

Appendix Figure S3. Phenotypic analysis of *abo1-Pk* and *abo1-GFP* strains.
A. The indicated strains were grown to mid log phase, subjected to five-fold serial dilution and spotted onto YES5 plates and incubated at 37°C for 2 days. **B.** As for A, except cells were spotted on YE5S plates lacking adenine and incubated at 30°C for 3-4 days. Data is representative of two biological repeats.

Appendix Table S1. Strains used in this study.

Strain	Genotype	Ref / Source
NT5	*h^- ade6-216 leu1-32 ura4-D18*	Lab stock
SW759	*h^+ ade6-216 leu1-32 ura4-D18 abo1::kanMX*	[1]
SW760	*h^+ ade6-216 leu1-32 ura4-D18 abo2::kanMX*	[1]
SW232	*h^+ ade6-210 leu1-32 ura4-DS/E imr1R(NcoI)::ura4$^+$*	[2]
SW873	*h^+ ade6-210 leu1-32 ura4- imr1R(NcoI)::ura4$^+$ abo1::kanMX*	This study
SW874	*h^+ ade6-210 leu1-32 ura4- imr1R(NcoI)::ura4$^+$ abo1::kanMX*	This study
SW875	*h^+ ade6-210 leu1-32 ura4- imr1R(NcoI)::ura4$^+$ abo2::kanMX*	This study
SW876	*h^+ ade6-210 leu1-32 ura4- imr1R(NcoI)::ura4$^+$ abo2::kanMX*	This study
FY4811	*h^+ ade6-210 leu1-32 ura4-DS/E otr1R(SphI)::ura4$^+$*	[2]
SW844	*h^+ ade6-210 leu1-32 ura4-DS/E otr1R(SphI)::ura4$^+$ abo1::kanMX*	This study
SW845	*h^+ ade6-210 leu1-32 ura4-DS/E otr1R(SphI)::ura4$^+$ abo1::kanMX*	This study
SW846	*h^- ade6-210 leu1-32 ura4-DS/E otr1R(SphI)::ura4$^+$ abo2::kanMX*	This study
SW847	*h^+ ade6-210 leu1-32 ura4-DS/E otr1R(SphI)::ura4$^+$ abo2::kanMX*	This study
PG1672	*mat1-PΔ17::LEU2 mat3-M(EcoRV)::ade6 ade6-M210 leu1-32 ura4-D18*	[3]
SW840	*mat1-PΔ17::LEU2 mat3-M(EcoRV)::ade6 ade6-M210 leu1-32 ura4-D18 abo1::kanMX*	This study
SW841	*mat1-PΔ17::LEU2 mat3-M(EcoRV)::ade6 ade6-M210 leu1-32 ura4-D18 abo1::kanMX*	This study
SW842	*mat1-PΔ17::LEU2 mat3-M(EcoRV)::ade6 ade6-M210 leu1-32 ura4-D18 abo2::kanMX*	This study
SW843	*mat1-PΔ17::LEU2 mat3-M(EcoRV)::ade6 ade6-M210 leu1-32 ura4-D18 abo2::kanMX*	This study
972	*h^-*	Lab stock
HM463	*h^- abo1::kanMX*	This study
HM466	*h^- abo2::kanMX*	This study
PY1798	*h^- ade6-210 leu1-32 ura4-DS/E arg3-D4 his3-D1 clr4::kanMX*	[4]
A8890	*h^+ ade6-210 leu1-32 ura4-D18 arg3-D4 his3-D1 abo1-GFP(kanMX)*	This study
SW865	*h^- ade6-M216 leu1-32 ura4-D18 abo1-PK(ura4$^+$)*	This study
A5259	*h^- spt16-18(kanMX) leu1-32*	[5]
EL238	*h^- ade6-210 leu1-32 ura4-DS/E arg3-D4 his3-D1 pob3::natMX*	[6]
EL313	*h^+ ade6-M210 arg3-D4 his3-D1 leu1-32 ura4-DS/E spt16-L-GFP(kanMX)*	[6]

EL130	*h⁻ otr1R(Sph1):ura4⁺ arg3-D4 his3-D1 leu1-32 ura4-DS/E pob3-L-GFP(kanMX)*	[6]
CsG338	*h⁺ ade6-216 ura4-D18 leu1-32 his3-D1 spt16-L-GFP(kanMX) abo1-PK(ura4⁺)*	This study
CsG351	*h+/- ade6-210 otr1R(Sph1):ura4⁺ arg3-D4 leu1-32 ura4⁻ pob3-L-GFP(kanMX) abo1-PK(ura4⁺)*	This study
CsG352	*h+/- ade6-210 leu1-32 ura4-D18 arg3-D4 abo1-GFP(kanMX) pob3::natMX*	This study
CsG360	*h+/- leu1-32 ura4⁻D18 arg3-D4 abo1-GFP(kanMX) spt16-18(kanMX)*	This study
CsG345	*h⁻ ade6-210 leu1-32 ura4-D18 / DS/E his3-D1 arg3-D3 spt16-L-GFP(kanMX) abo1::kanMX*	This study
CsG346	*h⁻ leu1-32 ura4-D18 / DS/E his3-D1 arg3-D3 pob3-L-GFP(kanMX) abo1::kanMX*	This study
CsG363	*h+/- ade6-210 leu1-32 ura4-D18 abo1-GFP(kanMX) swi6::kanMX*	This study
HM136	*h⁻ ade6-216 leu1-32 ura4-D18 Tf2-6::lacZ(ura4⁺)*	[7]
HM361	*h⁻ ade6-216 leu1-32 ura4-D18 Tf2-6::lacZ(ura4⁺) abo1::kanMX*	This study
HM353	*h⁻ ade6-216 leu1-32 ura4-D18 Tf2-6::lacZ(ura4⁺) abo2::kanMX*	This study
HM60	*h⁻ ade6-216 leu1-32 ura4-D18 chrmII::Tf1-lacZ(ura4⁺)*	This study
HM362	*h⁻ ade6⁻ leu1-32 ura4-D18 chrmII::Tf1-lacZ(ura4⁺) abo1::kanMX*	This study
HM372	*h⁻ ade6⁻ leu1-32 ura4-D18 chrmII::Tf1-lacZ(ura4⁺) abo2::kanMX*	This study
SW700	*h⁻ ade6-210 leu1-32 ura4-D18 hip1::ura4⁺*	[8]
SW877	*h⁻ ade6-210 leu1-32 ura4-D18 Ch16 R-23*	[8]
SW881	*h⁻ ade6-210 leu1-32 ura4-D18 abo1::kanMX Ch16 R23*	This study
SW882	*h⁻ ade6-210 leu1-32 ura4-D18 abo2::kanMX Ch16 R23*	This study
SW886	*h90 ade6-210 leu1-32 ura4-D18*	Lab stock
SW889	*h90 ade6-210 leu1-32 ura4-D18 abo1::kanMX*	This study
SW887	*h90 ade6-210 leu1-32 ura4-D18 abo2::kanMX*	This study
SW890	*h⁻ ade6-216 leu1-32 ura4- otr1R(SphI)::ura4⁺ ars1(MluI)::pRep81XGFPSwi6(LEU2) abo1::kanMX*	This study
PY6627	*h⁻ ade6-216 leu1-32 ura4- otr1R(SphI)::ura4⁺ ars1(MluI)::pRep81XGFPSwi6(LEU2)*	This study
PY6629	*h⁻ade6-216 leu1-32 ura4- otr1R(SphI)::ura4⁺ ars1(MluI)::pRep81XGFPSwi6(LEU2) clr4::kanMX*	This study
AW006	*h⁺ ade6-216 leu1-32 ura4-D18 abo1::natMX*	This study
AW057	*h-/+ ade6⁻ leu1-32 ura4-D18 abo1::natMX pob3::kanMX*	This study
AW058	*h⁺ ade6-216 leu1-32 ura4-D18 nhp6::kanMX*	[1]

AW039	$h^{-/+}$ *ade6*$^{-}$ *leu1-32 ura4-D18 abo1::natMX nhp6::kanMX*	This study
AW056	h^{+} *ade6-216 leu1-32 ura4-D18 pob3::kanMX*	[1]

References

1. Kim DU et al (2010) *Nature biotechnology*. **28**: 617-623.
2. Allshire RC et al (1995) *Genes Dev*. **9**: 218-233.
3. Thon G, Bjerling KP, Nielsen IS (1999) *Genetics*. **151**: 945-963.
4. Debeauchamp JL et al (2008) *Mol Cell Biol*. **28**: 2154-2166.
5. Choi ES et al (2012) *PLoS Genet*. **8**: e1002985.
6. Lejeune E et al (2007) *Curr Biol*. **17**: 1219-1224.
7. Anderson HE et al (2009) *Mol Cell Biol*. **29**: 5158-5167.
8. Blackwell C et al (2004) *Mol Cell Biol*. **24**: 4309-4320.
