# Supplementary material for: Abo1, a conserved bromodomain AAA‐ATPase, maintains global nucleosome occupancy and organisation
Source: EMBO Rep. 2015 Nov 18;17(1):79–93. doi: 10.15252/embr.201540476 (PMC4718406; doi:10.15252/embr.201540476)
Supplement: Supplementary file 2 — Expanded View Figures PDF [file EMBR-17-079-s002.pdf]

Expanded View Figures

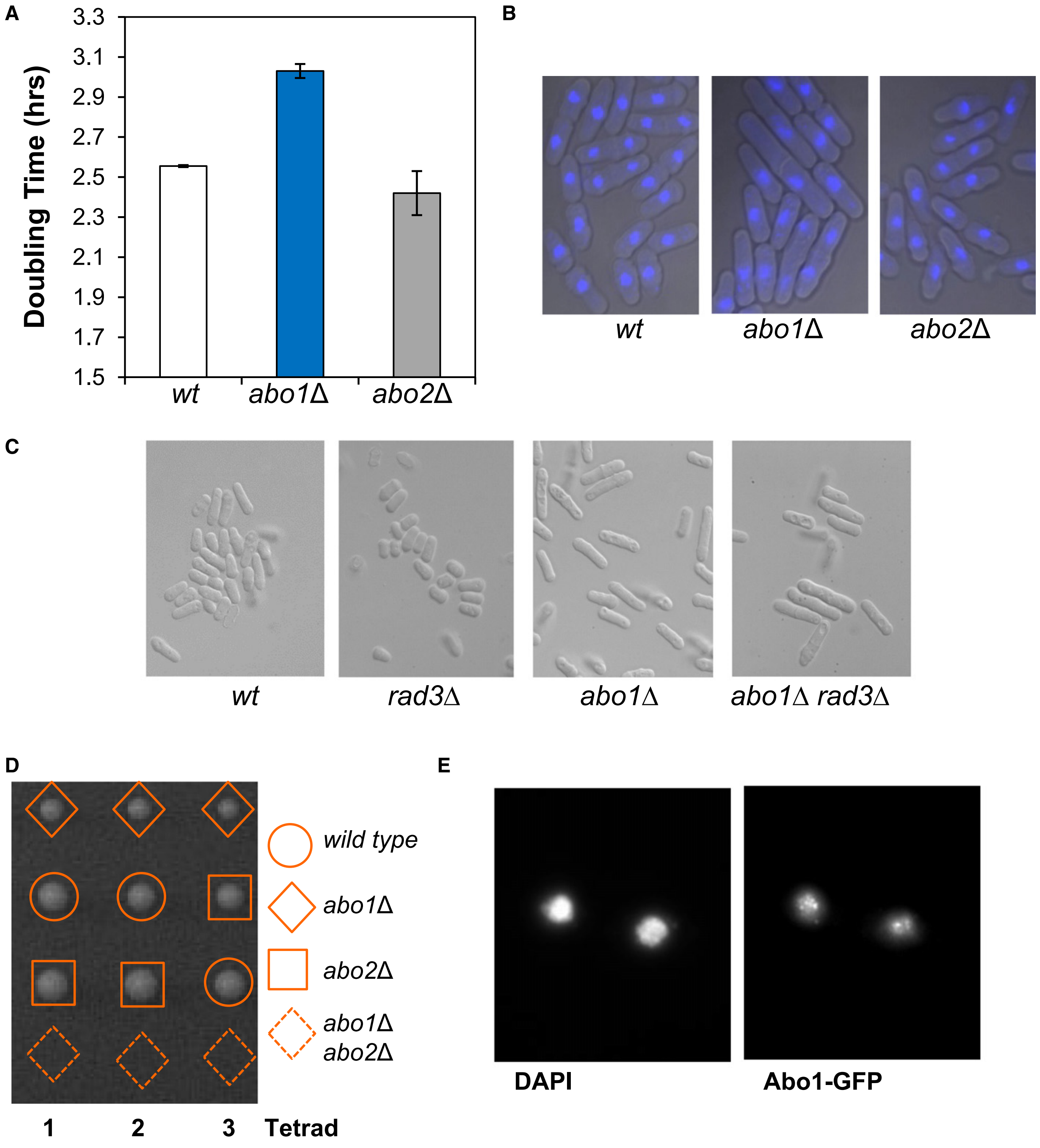

Figure EV1.

© 2015 The Authors

Figure EV1. Phenotypes associated with deletion of *abo1*⁺.

A Doubling time was estimated by measuring the OD_{595} of the indicated strains grown in YE5S medium at 30°C. Data are the mean of two independent biological repeats, and error bars denote the range of the data.

B *abo1Δ* cells have an elongated morphology. Microscopic analysis of DAPI-stained cells. Data are representative of three biological repeats.

C The elongated morphology of *abo1Δ* cells is independent of the ATR checkpoint kinase, Rad3. Microscopic analysis of the indicated strain. Images are representative of duplicate experiments.

D An *abo1Δ abo2Δ* double mutant strain is not viable. Tetrad dissection of a genetic cross between *abo1Δ* and *abo2Δ*. Colonies arising from the spores of three asci are shown. Data are representative of three biological repeats.

E Subcellular localisation of Abo1. Cells expressing Abo1-GFP were stained with DAPI and visualised using fluorescence microscopy. Data are representative of two biological repeats.

Figure EV2. Paired-end mode chromatin-seq of *abo1Δ*.

A Ethidium-stained agarose gel separations of the two DNA pools (Biorep1 and Biorep2) extracted from MNase-digested *S. pombe* chromatin and used for chromatin sequencing in this study.

B Frequency distributions of paired-read end-to-end size values after chromatin-seq of DNAs shown in (A). Note that increased MNase digestion used for Biorep2 samples shifts end-to-end size values downwards as expected.

C Nucleosome positions in wild-type (Biorep1) cells were defined as the locations of 150 ± 30 bp (nucleosome) size class particle frequency peak summits (frequency value > 25). This simple heuristic procedure identifies 60,658 putative positioned nucleosomes in the *S. pombe* genome. The nucleosome size class particle frequency distributions centred on and surrounding (± 1,200 bp) these positions were then smoothed using an Epanechnikov kernel density estimate (to match that of a previously published data set (Gene Expression Omnibus GSE40451 [30]), summed and normalised to the average frequency value occurring in the ± 1,200 bp window, for each of the data sets. These cumulative distributions reveal the average nucleosome organisation surrounding positioned nucleosomes in the genome of each cell type. Three pair-wise comparisons are shown. The nucleosome distribution from wild-type Biorep1 overlaps with that in a previously published wild-type data set (Gene Expression Omnibus GSE40451 [30]), confirming that our nucleosome mapping method yields similar results to those obtained using other technology. The *abo1Δ* mutant nucleosome distributions observed in Bioreps 1 and 2 both show a lower peak height and higher trough depth than the corresponding wild-type. The wavelength of the peak pattern is shown and is equal to the known *S. pombe* nucleosome repeat length.

D Genome browser plot of wild-type nucleosome occupancy data set (Gene Expression Omnibus GSE40451 [30]) plotted in relation to the 150 ± 30 bp paired-read mid-point frequency data obtained in this study (smoothed using an Epanechnikov kernel density estimate). Peak positions match between the two wild-type data sets, confirming that the 150 ± 30 bp class of paired sequence reads accurately represents nucleosomal species from chromatin. Nucleosome positions in the wild-type Biorep1 data set defined by our heuristic peak marking procedure are shown as “marked nucleosomes”.

 © 2015 The Authors

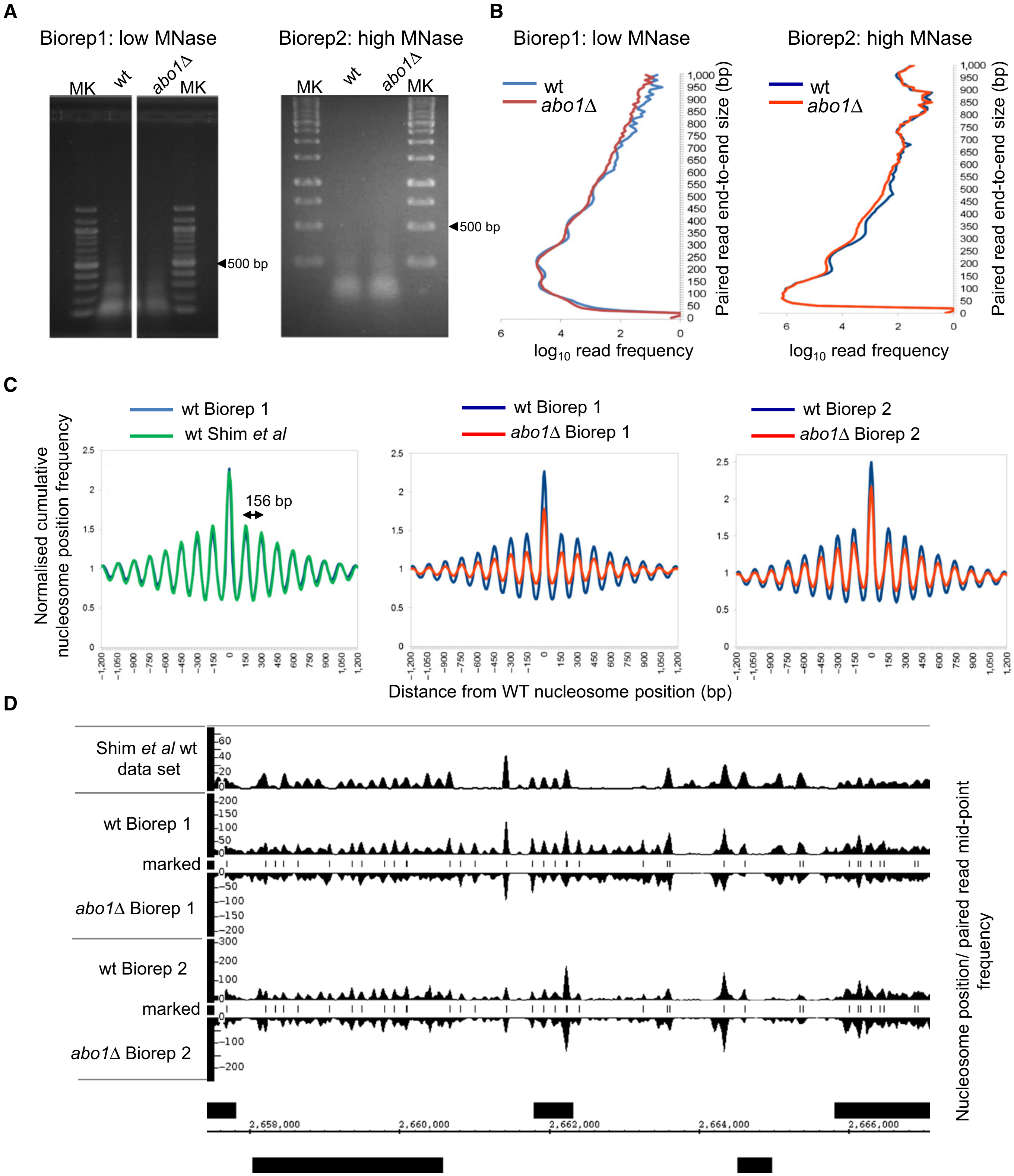
A
Biorep1: low MNase
Biorep2: high MNase
MK
wt
abo1Δ
MK
500 bp
B
Biorep1: low MNase
Biorep2: high MNase
wt
abo1Δ
Paired read end-to-end size (bp)
log10 read frequency
C
wt Biorep 1
wt Shim et al
156 bp
wt Biorep 1
abo1Δ Biorep 1
wt Biorep 2
abo1Δ Biorep 2
Normalised cumulative nucleosome position frequency
Distance from WT nucleosome position (bp)
D
Shim et al wt data set
wt Biorep 1
marked
abo1Δ Biorep 1
wt Biorep 2
marked
abo1Δ Biorep 2
2,658,000
2,660,000
2,662,000
2,664,000
2,666,000
Nucleosome position/ paired read mid-point frequency

Figure EV2.

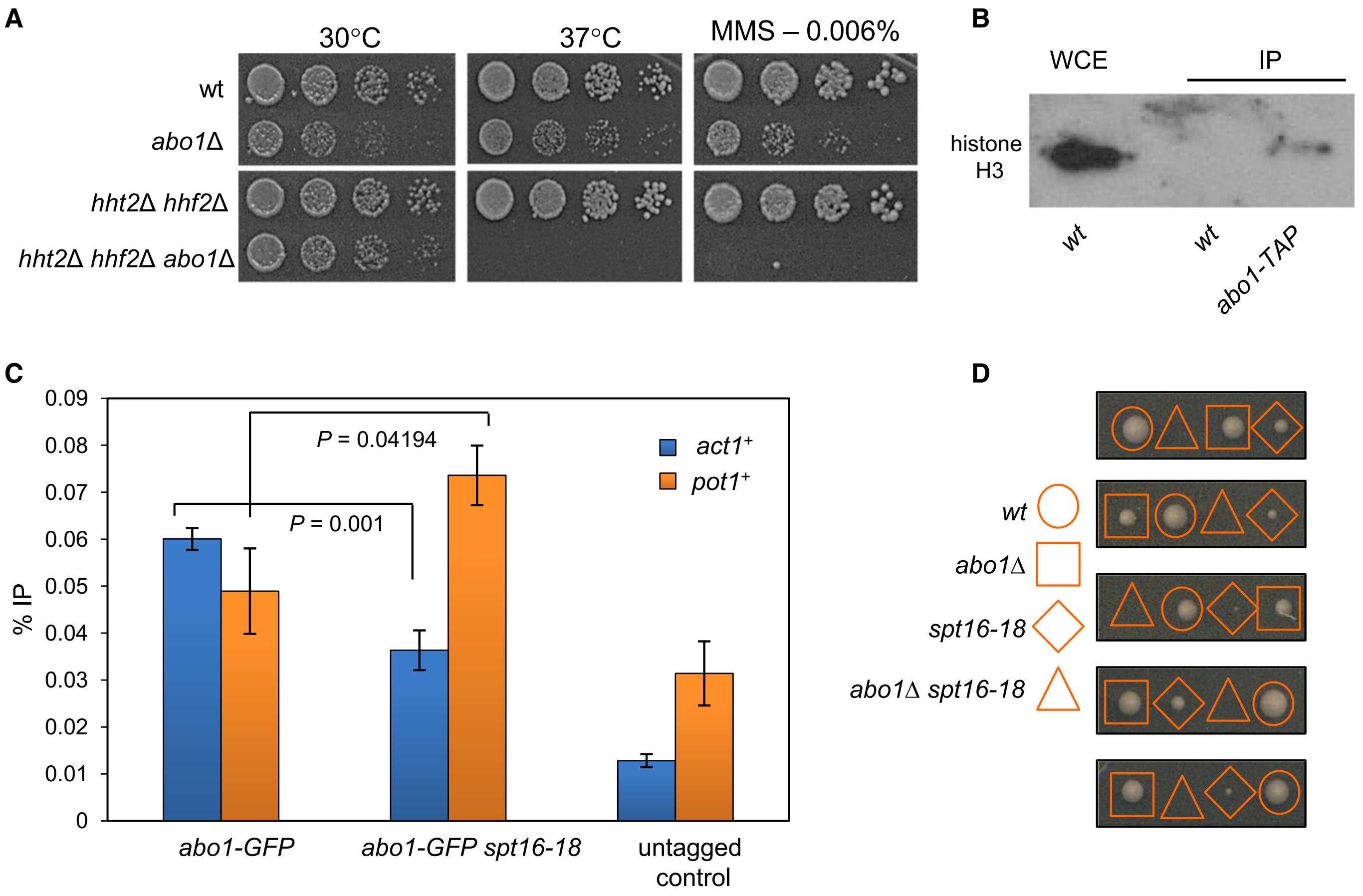

Figure EV3. Abo1 physically and genetically interacts with histones and FACT.

A Deletion of the histone H3–H4 gene pair *hht2⁺–hhf2⁺* exacerbates phenotypes associated with deletion of *abo1⁺*. The indicated strains were grown to mid log phase, subjected to five-fold serial dilution and spotted onto YES5 plates supplemented as indicated. Plates were incubated for 2 days (37°C) or 3–5 days (30°C). Images are representative of three biological repeats. All strains were present on the same agar plates.

B Whole-cell extracts prepared from the indicated strains were partially purified using IgG sepharose and analysed by Western blotting. Data are representative of two biological repeats.

C ChIP–qPCR analysis was used to detect Abo1-GFP enrichment at the indicated loci in mid log-phase cells grown at 30°C. Data are the mean of three independent repeats, and error bars denote ± SEM. *P*-values were calculated using a two-tailed unpaired *t*-test. GFP-tagged strains exhibit significant enrichment ($P < 0.05$) at both loci relative to the untagged control.

D Tetrad dissection of a genetic cross between *abo1Δ* and *spt16-18*. The genotypes of colonies arising from the spores of five asci are shown. Analysis of a total of 28 tetrads from two independent genetic crosses failed to identify a viable double mutant strain.

 © 2015 The Authors

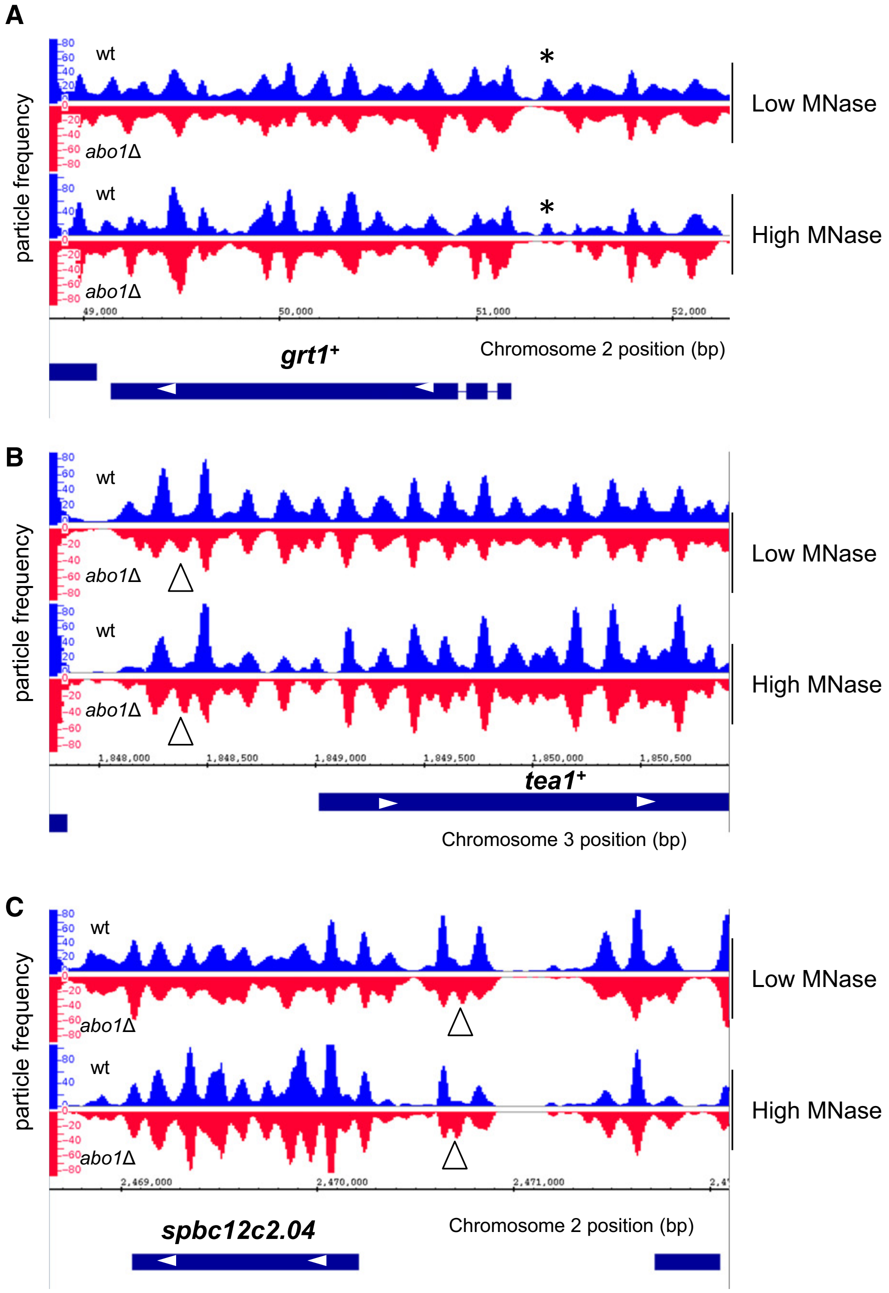

Figure EV4. Abo1 is required for the organisation of chromatin in promoters.

A–C Nucleosome (150 ± 30 bp size class) read profile over the indicated chromosomal loci. Data from low MNase (biorep1) are shown in the top panel and data from high MNase (biorep2) in the bottom panel. Missing or additional peaks in the *abo1Δ* background are marked with asterisks and triangles, respectively.

© 2015 The Authors

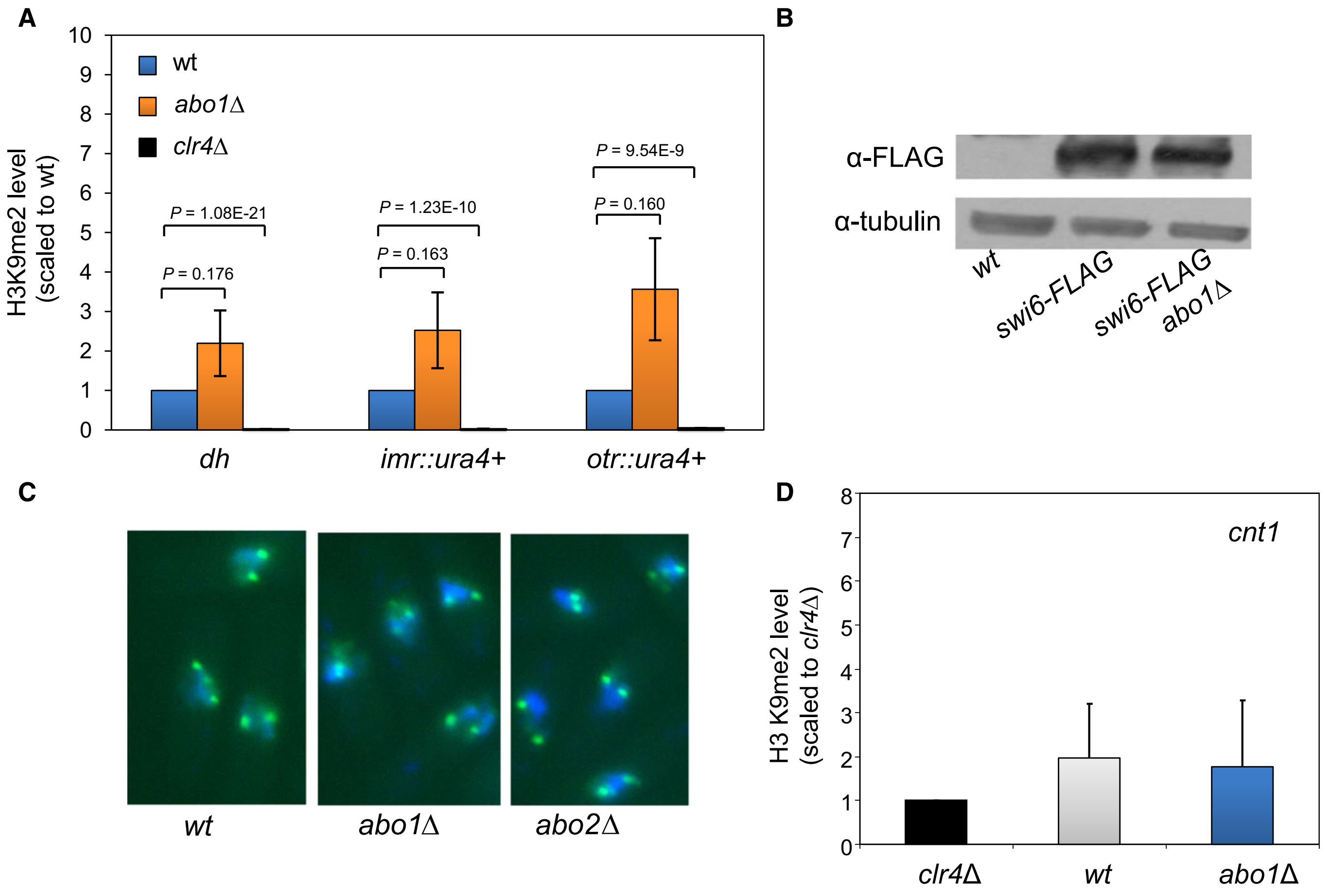

Figure EV5. Loss of Abo1 does not reduce H3K9me2 or Swi6/HP1.

A H3K9me2 levels at *dh* repeats and a centromeric *ura4*$^+$ marker gene were determined by ChIP–qPCR. H3K9me2 enrichment relative to *adh1*$^+$ was determined and levels scaled to wild-type. Data are the mean of three independent biological replicates, and error bars represent ± SEM. *P*-values were calculated using a two-tailed unpaired *t*-test.

B Whole-cell extracts prepared from the indicated strains were analysed by Western blotting with the indicated antibodies. Data are representative of two independent biological repeats.

C Cell expressing GFP-Swi6 were stained with DAPI and visualised using fluorescence microscopy. Data are representative of two independent biological repeats.

D Loss of Abo1 does not result in the inappropriate spread of H3K9me2 into the centromeric central core. ChIP analysis of H3K9me2 levels at central core (*cnt*) sequences of chromosome 1. H3K9me2 enrichment relative to *adh1*$^+$ was determined and levels scaled to the *clr4*Δ (-H3 K9me2) mutant. Data are the mean of three independent ChIP experiments, and error bars are ± SEM.

 © 2015 The Authors
