# Supplementary material for: Abo1, a conserved bromodomain AAA‐ATPase, maintains global nucleosome occupancy and organisation
Source: EMBO Rep. 2015 Nov 18;17(1):79–93. doi: 10.15252/embr.201540476 (PMC4718406; doi:10.15252/embr.201540476)
Supplement: Supplementary file 5 — Source Data for Figure 2 [file EMBR-17-079-s004.pptx]

## Slide 1
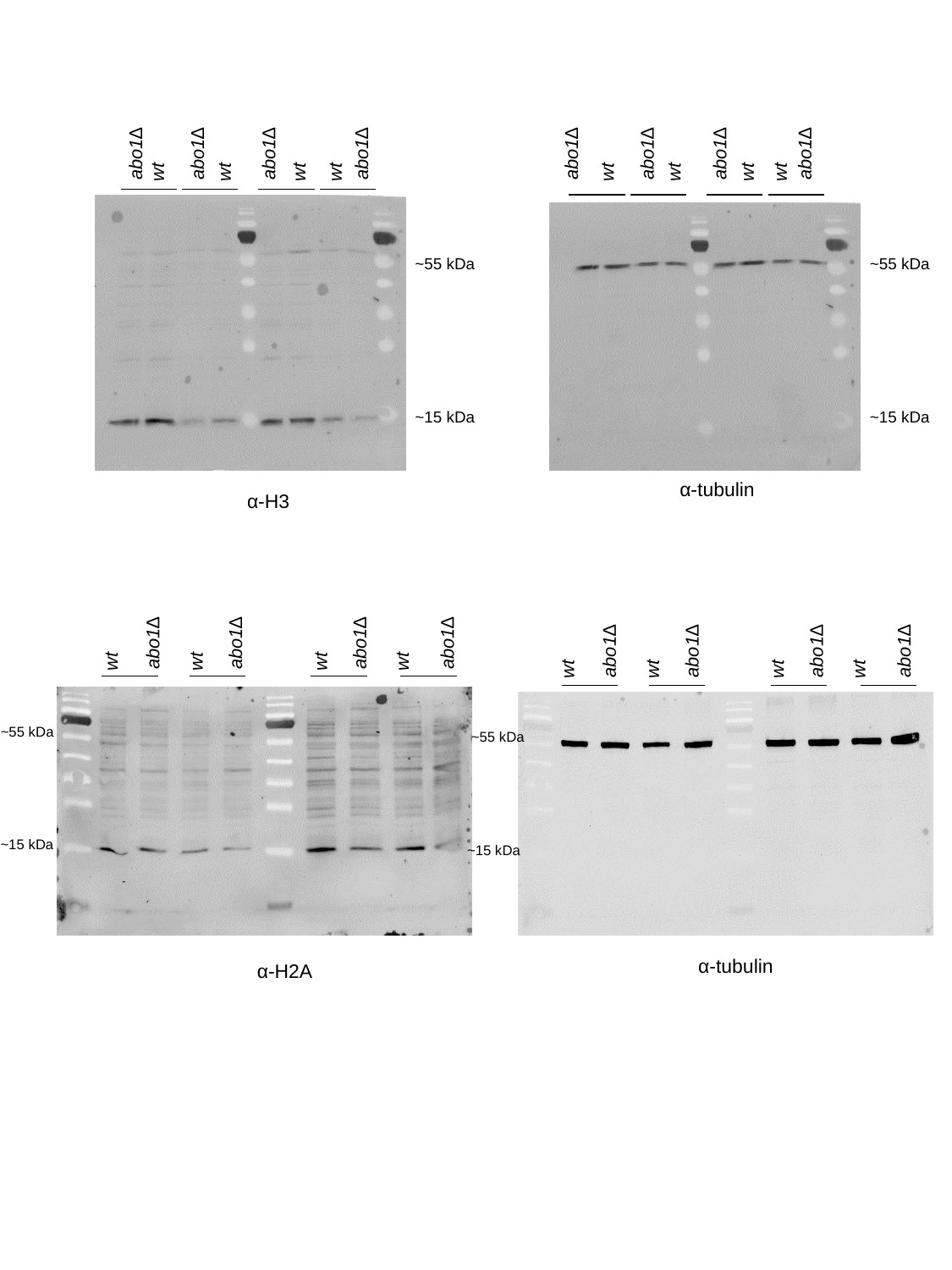

abo1∆
abo1∆
abo1∆
abo1∆
abo1∆
abo1∆
abo1∆
abo1∆
wt
wt
wt
wt
wt
wt
wt
wt
~55 kDa
~55 kDa
~15 kDa
~15 kDa
α-tubulin
α-H3
abo1∆
abo1∆
abo1∆
abo1∆
wt
wt
wt
wt
abo1∆
abo1∆
abo1∆
abo1∆
wt
wt
wt
wt
~55 kDa
~55 kDa
~15 kDa
~15 kDa
α-tubulin
α-H2A
