# Supplementary figures and images for: Abo1, a conserved bromodomain AAA‐ATPase, maintains global nucleosome occupancy and organisation
Source: EMBO Rep. 2015 Nov 18;17(1):79–93. doi: 10.15252/embr.201540476 (PMC4718406; doi:10.15252/embr.201540476)

## Slide 1
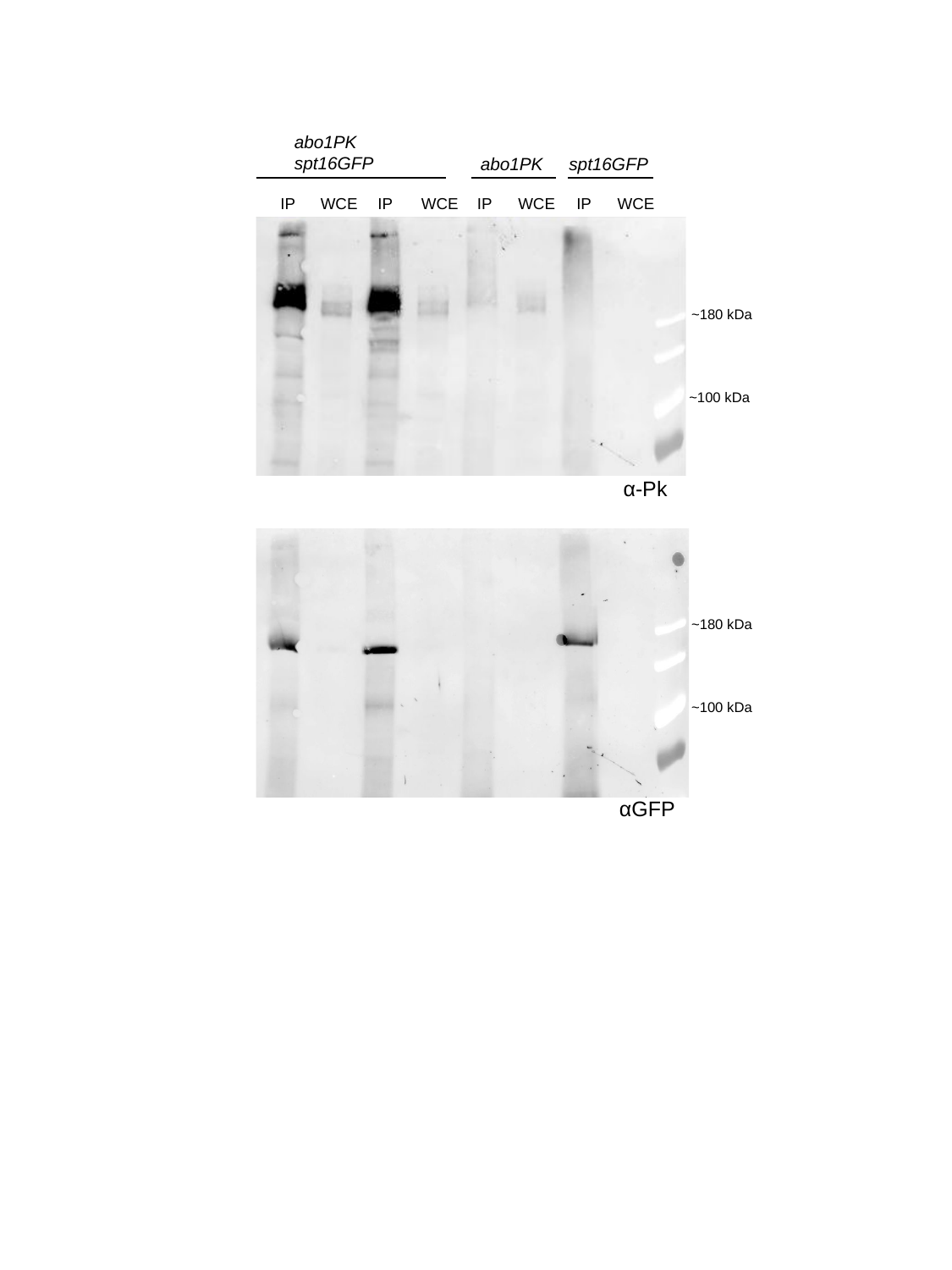

abo1PK
spt16GFP
abo1PK
spt16GFP
IP
WCE
IP
WCE
IP
WCE
IP
WCE
~180 kDa
~100 kDa
α-Pk
~180 kDa
~100 kDa
αGFP

Supplement: Supplementary file 6 — Source Data for Figure 3 [file EMBR-17-079-s005.zip › Source Data Fig3/SourceDataForFig3A.pptx]

## Slide 1
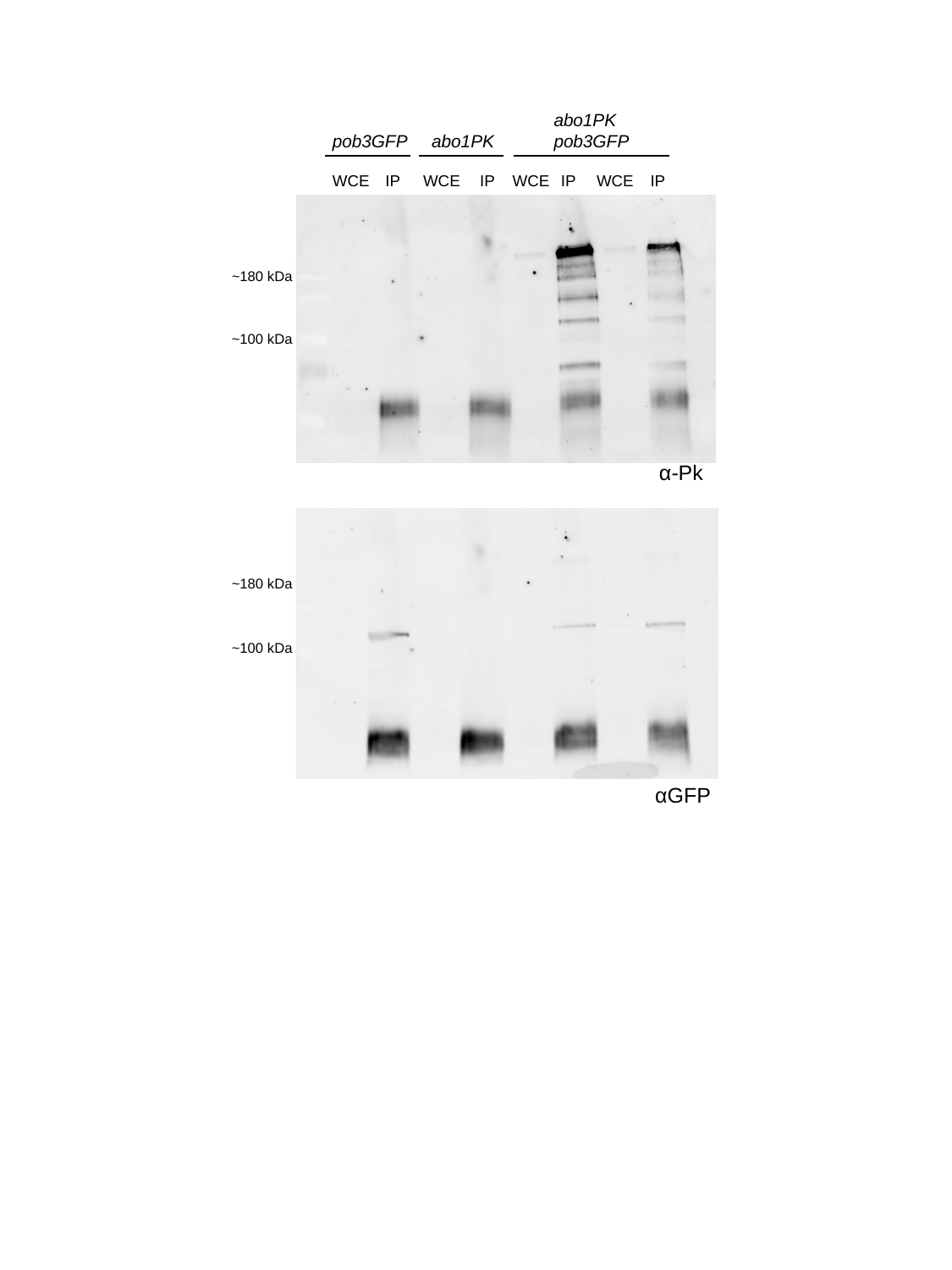

abo1PK
pob3GFP
pob3GFP
abo1PK
WCE
IP
WCE
IP
WCE
IP
WCE
IP
~180 kDa
~100 kDa
α-Pk
~180 kDa
~100 kDa
αGFP

Supplement: Supplementary file 6 — Source Data for Figure 3 [file EMBR-17-079-s005.zip › Source Data Fig3/SourceDataForFig3B.pptx]

## Slide 1
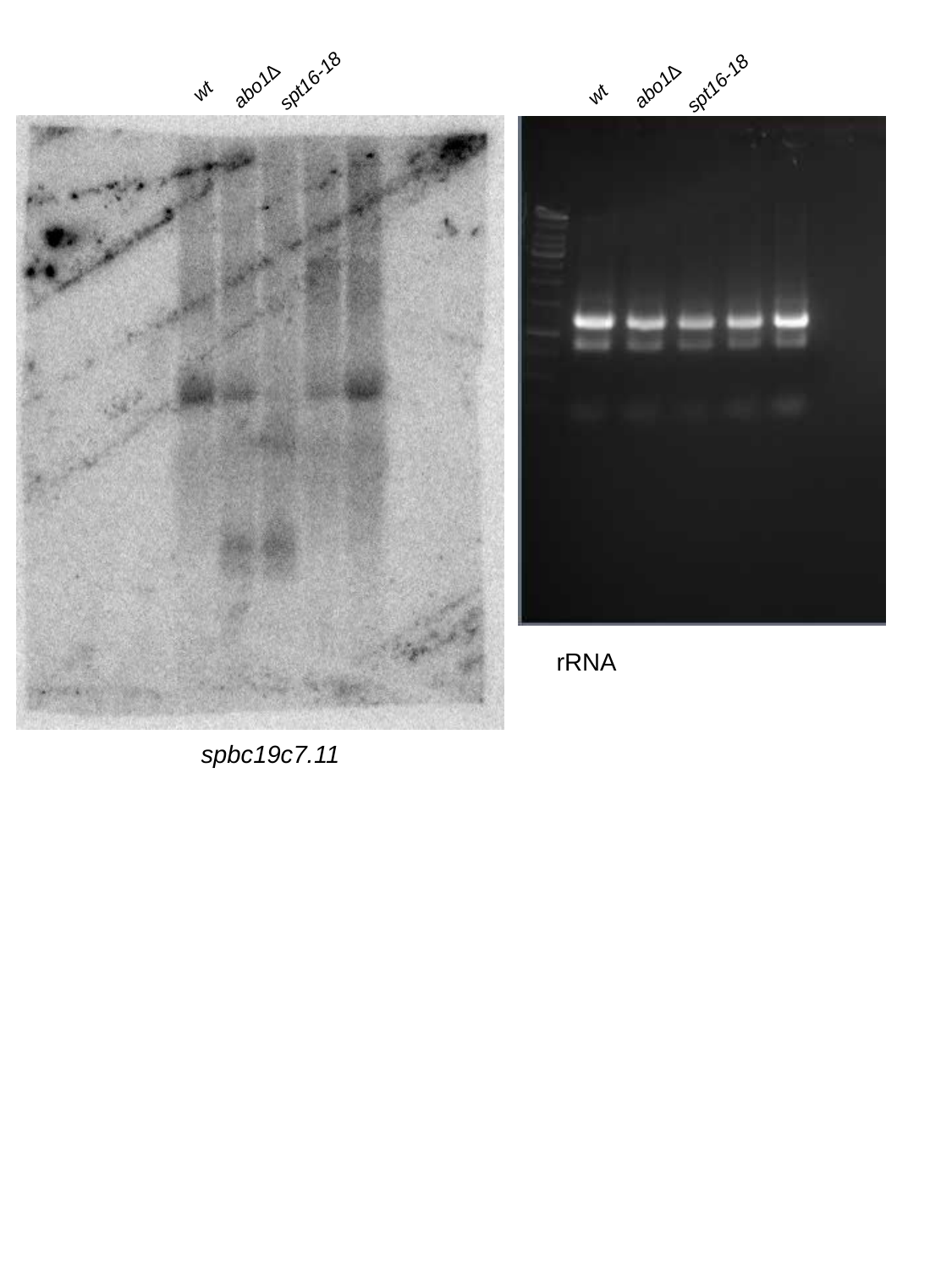

spt16-18
spt16-18
abo1∆
abo1∆
wt
wt
rRNA
spbc19c7.11

Supplement: Supplementary file 6 — Source Data for Figure 3 [file EMBR-17-079-s005.zip › Source Data Fig3/SourceDataForFig3C.pptx]
