# Supplementary material for: Abo1, a conserved bromodomain AAA‐ATPase, maintains global nucleosome occupancy and organisation
Source: EMBO Rep. 2015 Nov 18;17(1):79–93. doi: 10.15252/embr.201540476 (PMC4718406; doi:10.15252/embr.201540476)
Supplement: Supplementary file 4 — Review Process File [file EMBR-17-079-s006.pdf]

EMBO
reports

Manuscript EMBO-2015-40476

Abo1, a conserved bromodomain AAA-ATPase, maintains global nucleosome occupancy and organization

Csenge Gal, Heather E. Murton, Lakxmi Subramanian, Alex J. Whale, Karen M. Moore, Konrad Paszkiewicz, Sandra Codlin, Jürg Bähler, Kevin M. Creamer, Janet F. Partridge, Robin C. Allshire, Nicholas A. Kent, and Simon K. Whitehall

Corresponding author: Simon K. Whitehall, University of Newcastle

Review timeline:

Submission date:	31 March 2015
Editorial Decision:	15 May 2015
Revision received:	24 September 2015
Editorial Decision:	13 October 2015
Revision received:	20 October 2015
Accepted:	26 October 2015

Editor: Esther Schnapp

No Peer Review Process File is available with this article, as the authors have chosen not to make the review process public in this case.

© European Molecular Biology Organization
